# Longitudinal associations between alcohol use, smoking, genetic risk scoring and symptoms of depression in the general population: a prospective 6-year cohort study

**DOI:** 10.1017/S0033291721002968

**Published:** 2023-03

**Authors:** N. de Boer, J. Vermeulen, B. Lin, J. van Os, M. ten Have, R. de Graaf, S. van Dorsselaer, M. Bak, B. Rutten, A. Batalla, S. Guloksuz, J. J. Luykx

**Affiliations:** 1 Department of Psychiatry, UMC Utrecht Brain Center, University Medical Center Utrecht, Utrecht University, Utrecht, The Netherlands; 2 Department of Psychiatry, Amsterdam UMC location AMC, University of Amsterdam, Amsterdam, The Netherlands; 3 Department of Translational Neuroscience, UMC Utrecht Brain Center, University Medical Center Utrecht, Utrecht University, Utrecht, The Netherlands; 4 Department of Psychiatry and Neuropsychology, School for Mental Health and Neuroscience, Maastricht University Medical Centre, Maastricht, The Netherlands; 5 Department of Psychosis Studies, Institute of Psychiatry, Psychology & Neuroscience, King's College London, London, UK; 6 Department of Epidemiology, Netherlands Institute of Mental Health and Addiction, Utrecht, The Netherlands; 7 FACT, Mondriaan Mental Health, Maastricht, The Netherlands; 8 Department of Psychiatry, Yale University School of Medicine, New Haven, CT, USA; 9 GGNet Mental Health, Apeldoorn, The Netherlands

**Keywords:** Alcohol use, general population, longitudinal cohort study, polygenic risk score, smoking, symptoms of depression

## Abstract

**Background:**

Alcohol consumption, smoking and mood disorders are leading contributors to the global burden of disease and are highly comorbid. Yet, their interrelationships have remained elusive. The aim of this study was to examine the multi-cross-sectional and longitudinal associations between (change in) smoking and alcohol use and (change in) number of depressive symptoms.

**Methods:**

In this prospective, longitudinal study, 6646 adults from the general population were included with follow-up measurements after 3 and 6 years. Linear mixed-effects models were used to test multi-cross-sectional and longitudinal associations, with smoking behaviour, alcohol use and genetic risk scores for smoking and alcohol use as independent variables and depressive symptoms as dependent variables.

**Results:**

In the multi-cross-sectional analysis, smoking status and number of cigarettes per day were positively associated with depressive symptoms (*p* < 0.001). Moderate drinking was associated with less symptoms of depression compared to non-use (*p* = 0.011). Longitudinally, decreases in the numbers of cigarettes per day and alcoholic drinks per week as well as alcohol cessation were associated with a reduction of depressive symptoms (*p* = 0.001–0.028). Results of genetic risk score analyses aligned with these findings.

**Conclusions:**

While cross-sectionally smoking and moderate alcohol use show opposing associations with depressive symptoms, decreases in smoking behaviour as well as alcohol consumption are associated with improvements in depressive symptoms over time. Although we cannot infer causality, these results open avenues to further investigate interventions targeting smoking and alcohol behaviours in people suffering from depressive symptoms.

## Introduction

Alcohol, tobacco use and mood disorders are prominent contributors to the global burden of diseases and are highly comorbid (Anthony, Warner, & Kessler, 1994; Degenhardt et al., 2018; Ezzati, Lopez, Rodgers, Vander Hoorn, & Murray, 2002; Kessler, Chiu, Demler, Walters, & Walters, 2005). Approximately 30% of individuals with major depressive disorder (MDD) have comorbid substance use disorders (SUD) (Davis et al., 2006). Vice versa, 30–50% of individuals in treatment for SUD have comorbid mood disorders (European Monitoring Centre for Drugs and Drug Addiction, 2015). Cross-sectional studies indicate a higher prevalence of smoking in people with MDD or with depressive symptoms and that symptoms of depression are more frequent in smokers than never- or former smokers (Anda et al., 1990; Glassman et al., 1990; Jamal, Willem Van der Does, Cuijpers, & Penninx, 2012). Moreover, the incidence of smoking rises as the level of depressive symptoms increases (Anda et al., 1990). Furthermore, alcohol abuse and dependence often coexist with depression (Flensborg-Madsen et al., 2009; Grant & Harford, 1995; Kessler et al., 2005, 1997).

Previous literature hints at a positive association between alcohol consumption, smoking and symptom levels of depression (Anda et al., 1990; Gilman & Abraham, 2001; Glassman et al., 1990; Grant & Harford, 1995; Pérez-Stable, Marín, Marín, & Katz, 1990; Pomerleau, Zucker, & Stewart, 2003; Sullivan, Fiellin, & O'Connor, 2005; Swendsen et al., 1998). Hypotheses explaining this association are based on genetic and environmental interactions, shared aetiology, direct causality or other mechanisms (Khantzian, 1997; O'Neil, Conner, & Kendall, 2011). First, smoking and alcohol use might induce depressive symptoms. Previous studies provide evidence that smoking cessation leads to a reduction of depressive symptoms (Jamal et al., 2012; Lechner, Sidhu, Cioe, & Kahler, 2019; Stepankova et al., 2017; Taylor et al., 2014). In addition, results of prospective studies show that alcohol abstinence reduces symptoms of depression (Brown et al., 1995; Brown & Schuckit, 1988; Liappas, Paparrigopoulos, Tzavellas, & Christodoulou, 2002). Second, longitudinal studies suggest that smoking is a potential risk factor for developing depression and that hazardous drinking and alcohol abuse as well as dependence increase the risk of depression (Boden & Fergusson, 2011; Boden, Fergusson, & Horwood, 2010; Breslau, Novak, & Kessler, 2004; Brown, Lewinsohn, Seeley, & Wagner, 1996; Fergusson, Boden, & Horwood, 2009; Gémes et al., 2019; Hasin, Goodwin, Stinson, & Grant, 2005). Other hypotheses for the association between smoking, alcohol use and depressive symptoms include misattribution or self-medication. The latter assumes that patients intend to alleviate certain symptoms (e.g. depression) or adverse effects from medication by using addictive substances (Bolton, Robinson, & Sareen, 2009; Breslau et al., 2004; Grant & Harford, 1995; Khantzian, 1997; Kuo, Gardner, Kendler, & Prescott, 2006; McKenzie, Olsson, Jorm, Romaniuk, & Patton, 2010). Misattribution states that people mislabel relief of withdrawal symptoms by drinking or smoking as positive effects of abuse (McKenzie et al., 2010; Wootton et al., 2020). Furthermore, other studies have argued that depressive symptoms lead to unhealthy coping such as alcohol abuse and dependence (Grant & Harford, 1995; Kuo et al., 2006). Evidently, the relation of the comorbidity between smoking, alcohol use and depressive symptoms remains unclear, which undermines the development of effective prevention strategies for mental and physical health issues in clinical practice.

Few large, longitudinal prospective studies have examined the associations between smoking, alcohol use and number of symptoms of depression in the general population (Cabello et al., 2017; Fergusson et al., 2009; Flensborg-Madsen et al., 2009). Importantly, previous studies have focused on a diagnosis of depression instead of symptoms of depression as outcome, resulting in a loss of power and of relevant clinical data as subsyndromal depressive symptoms may be associated with functional impairment as well (Cabello et al., 2017; Fergusson et al., 2009; Flensborg-Madsen et al., 2009; Gémes et al., 2019). Furthermore, suffering from an alcohol use disorder can render a clinical diagnosis of MDD less likely given the DSM-IV and DSM-5 exclusion criterion of substance use causing depression. Thus, depressive symptoms may be a relevant and clinically useful outcome measure to study the intricate relationships between nicotine and alcohol on the one hand and depressive symptoms on the other. Other limitations of previous studies investigating the association between tobacco use, drinking behaviour and depressive symptoms include the focus on alcohol use disorder instead of general alcohol drinking behaviour and smaller sample sizes (Fergusson et al., 2009; Flensborg-Madsen et al., 2009). Additionally, most studies have investigated the association between alcohol use and depression and between smoking and depression in separate cohorts. In sum, the relationships between quantitative measures of alcohol use and depressive symptoms on the one hand and smoking and depressive symptoms on the other hand have not yet been elucidated.

Based on previous literature, we hypothesized that smoking status and number of cigarettes per day are positively associated with depressive symptoms; and that smoking cessation is negatively associated with depressive symptoms. Similarly, for alcohol use, we hypothesized that alcohol use and severity are positively associated with depressive symptoms; and that alcohol cessation is negatively associated with depressive symptoms. We thus examined the multi-cross-sectional (cross-sectional relationship across multiple time points) and longitudinal associations between (changes in) smoking behaviour, alcohol use and number of depressive symptoms in a unique, large cohort from the general population. We used polygenic liabilities for these traits to corroborate the directionality of our findings.

## Methods

### Study population and procedure

Data were used from the Netherlands Mental Health Survey and Incidence Study-2 (NEMESIS-2). This stratified, a-selective study sample of the Dutch population included 6646 people between 18 and 64 years at the time of the baseline interview. The individuals of the sample were randomly selected from households in the Netherlands. Institutionalised individuals living in hostels, hospices or prisons or individuals with insufficient Dutch language proficiency were excluded. Further details concerning selection procedure and study design have been described by de Graaf et al. (de Graaf, ten Have, & van Dorsselaer, 2010). In the period of 2007–2015, three waves of data were derived from the study population of NEMESIS-2 and several outcome measures were assessed by trained interviewers. Follow-up rates were high with 3- and 6-year response rates of 79.8% (5303 people) and 69.5% (4618 people), respectively (Pries et al., 2018). Approval of an independent Medical Ethics Committee and written informed consent from all participants were obtained. The STROBE and TREND guidelines were followed to report this study (Des Jarlais, Lyles, Crepaz, & TREND Group, 2004; von Elm et al., 2007).

### Outcome measures

The dependent variable was the number of depressive symptoms using the Mental Health Inventory-3 (MHI-3) as primary outcome. The MHI-3 consists of three depression questions of the Mental Health Inventory (MHI-5), excluding two questions about anxiety symptoms that are included in the MHI-5 (see online Supplementary material for additional information regarding dependent variables). Previous studies have shown that both the MHI-3 and MHI-5 are valid instruments to identify individuals with depressive symptoms (Yamazaki, Fukuhara, & Green, 2005). Considering our focus on numeric depressive symptoms in the general population, and that some anxiety symptoms may be differentially associated with substance use (Groenman, Janssen, & Oosterlaan, 2017; Kaplow, Curran, Angold, & Costello, 2001), we chose to use the MHI-3 instead of the MHI-5 as primary outcome. The MHI-3 scores are 3–18 points, with lower scores indicating higher depressive symptoms. Subscales of the Short Form 36 Health Survey (SF-36) were used as sensitivity analysis (online Supplementary material) (Ware & Sherbourne, 1992). The SF-36 is a widely used instrument that assesses the level of mental and physical functioning in the past 4 weeks (Bos et al., 2018; McHorney, Ware, Rachel Lu, & Sherbourne, 1994). This self-report questionnaire consists of nine subscales each ranging from 0 to 100 points, representing poor to good functioning (McHorney et al., 1994). For our sensitivity analysis, the scores of three subscales of the SF-36 most closely associated with depressive symptoms (i.e. the vitality scale, the mental health scale or the MHI-5, and the emotional role functioning scale) were combined into one scale (total 0–300 points) as an outcome, indicating the number of depressive symptoms in the past 4 weeks, similarly to Bos et al. (2018). The score ranges from poor to good functioning, with low scores representing many depressive symptoms. Both primary (MHI-3) and sensitivity outcomes (SF-36) were measured at baseline (wave one), 3-year (wave two) and 6-year (wave three) follow-up.

### Independent variables

Alcohol use as binary trait and severity (drinks per week) and smoking status (smoking *v.* non-smoking) and severity (cigarettes per day) were the independent variables and measured at baseline, after 3 and 6 years. Smoking behaviour was assessed using self-designed questions, considering smoking status (binary) and daily smoking in the past month (categorized). The average number of cigarettes per day was categorized into six subgroups: <1 cigarette per week, >1 cigarette per week, 1–5, 6–10, 11–20 or >20 cigarettes per day (Cuijpers, Smit, ten Have, & de Graaf, 2007; de Graaf et al., 2010).

Alcohol consumption as a binary trait and severity (drinks per week) was measured using the CIDI 3.0 (American Psychiatric Association, 2000). Alcohol drinks per week were calculated with the frequency of drinking in the past 12 months and the number of glasses when drinking, similarly to Gémes et al. (2019) and Tuithof, Ten Have, van den Brink, Vollebergh, and de Graaf (2013). Drinking frequency was estimated by coding daily alcohol consumption as a weekly frequency of 7, almost every day as 5.5, 3–4 days per week as 3.5, 1–2 days per week as 1.5, 1–3 days per month as 0.5 and less than once per month as 0.125. The number of glasses per drinking day was multiplied by the estimated drinking frequency. Outliers were removed when above or below three standard deviations from the mean (removal of outliers below three standard deviations resulted in non-existing negative outcomes). Afterwards, drinks per week were categorized in four levels based on interquartile ranges (i.e. 0–1.5, 1.5–3.5, 3.5–10.5, 10.5–84.0 drinks per week). In this study, 10.5–84.0 drinks per week was classified as heavy drinking, 3.5–10.5 drinks per week as moderate drinking and 1.5–3.5 drinks per week as light drinking. In validation analyses, drinks per week were recategorized for women (i.e. 0–1, 1–14, 14–84 drinks per week) and men (i.e. 0–1, 1–21, 21–84 drinks per week), based on the current definitions for excessive alcohol use in the Netherlands (i.e. >14 standard units per week for women and >21 for men) (De Staat van Volksgezondheid en Zorg, 2020).

For longitudinal analyses, changes in smoking and alcohol use behaviour (yes/no) and changes in drinks per week and cigarettes per day [both binary change (increase/decrease) and per level] were determined and calculated between 0–3 and 3–6-year follow-up, similarly to previous studies (Vermeulen et al., 2018; Vermeulen et al., 2019). For example, someone who smoked at baseline and stopped smoking at 3-year follow-up and smoked again at 6-year follow-up was registered as ‘quit smoking’ between 0 and 3-year follow-up and ‘started smoking’ between 3 and 6-year follow-up. A reduction of 10.5–84.0 drinks per week to 1.5–3.5 drinks per week between baseline and 3-year follow-up was described as ‘decrease’ in binary models or ‘decrease of 2 levels’ in models per level. Furthermore, the change in the number of depressive symptoms was calculated by the difference in total scores between 0 and 3-year follow-up and 3 and 6-year follow-up.

Covariates were age, gender, treatment for any psychiatric disorder or negative life events in the previous 12 months, and cannabis dependency/abuse – all measured as binary traits except for age; and education level, which was categorized into either primary, lower secondary, higher secondary or higher professional education. Of these, education level and treatment for a psychiatric disorder were assessed using self-designed questions, while negative life events were measured using questions based on the ‘Brugha Life events section’ and cannabis dependency/abuse was determined by the CIDI 3.0 (Brugha & Cragg, 1990). All covariates were determined *a priori* based on previous studies demonstrating significant associations or using similar covariates with regards to smoking, alcohol use and depressive symptoms (Chaiton, Cohen, Rehm, Abdulle, & O'loughlin, 2014; Fergusson et al., 2009; Flensborg-Madsen et al., 2009; Gémes et al., 2019; Hooshmand, Willoughby, & Good, 2012; Kotov, Guey, Bromet, & Schwartz, 2010; Troisi, Pasini, Saracco, & Spalletta, 1998; Wilkinson, Halpern, & Herring, 2016). No other substance use than cannabis was included as a covariate given the higher prevalence (European Monitoring Centre for Drugs and Drug Addiction, 2006).

For the purpose of genetic analyses, polygenic risk scores (PRS) were computed in all genotyped participants for three phenotypes – alcohol drinks per week, smoking initiation and cigarettes per day – for 12 *p* value thresholds (0.5, 0.4, 0.3, 0.2, 0.1, 0.05, 5 × 10^−3^, 5 × 10^−4^, 5 × 10^−5^, 5 × 10^−6^, 5 × 10^−7^, 5 × 10^−8^). PRS estimate the genetic liability of an individual for specific traits or diseases (Choi, Mak, & O'Reilly, 2020). See online Supplementary material for extensive details regarding PRS analyses (quality control, imputation and principle component analyses).

### Statistical analysis

First, cross-sectional baseline comparisons between (1) smoking *v.* non-smoking and (2) alcohol *v.* non-alcohol use were conducted using unpaired *t* test and χ^2^ test at baseline. Second, linear mixed-effects models corrected for age and gender were applied for multi-cross-sectional analysis followed by longitudinal analysis of the associations between change in alcohol use, smoking and change in the number of depressive symptoms over a period of 6 years. To that end, (change in) smoking status (smoker or non-smoker) or (change in) alcohol status (drinker or non-drinker), wave, age and gender were included as fixed effects. Intercepts for respondents and by-subject random slopes for time were entered as random effects. To gain insight into dose–response associations, the number of cigarettes per day and drinks per week were then entered as predictors. Third, in our first sensitivity analysis, we replaced the MHI-3 with the SF-36 as outcome (see above under ‘Outcome measures’). Fourth, in our second sensitivity analysis, we corrected for all covariates listed under ‘Independent variables’ for both the MHI-3 and SF-36. Fifth, to examine whether associations of smoking and of alcohol use with the number of depressive symptoms were independent of each other, alcohol use and drinks per week were entered as additional covariates to the second sensitivity analysis model for smoking status/cigarettes per day and smoking status and cigarettes per day for alcohol use/drinks per week. At last, we validated our findings for drinks per week and repeated the analyses using different thresholds for women and men (see ‘Independent variables’).

All variables were added in a forward approach. People were included for multi-cross-sectional analysis whenever data regarding smoking or alcohol use and number of depressive symptoms were available for at least one of three measurements (at baseline, after 3 years or after 6 years) due to valid estimation calculation of mixed models. Participants were included for longitudinal analysis whenever data on change in smoking or alcohol use and change in number of depressive symptoms were available for at least two measurements with a minimum follow-up period of 3 years.

Finally, linear mixed-effects models were used for genetic analyses of the associations between PRS for smoking initiation, cigarettes per day and alcohol drinks per week and depressive symptoms. PRS models were adjusted for three ancestry principal components (PC1, PC2 and PC3) (Guloksuz et al., 2020). PRS for either smoking initiation, cigarettes per day or alcohol drinks per week using one of the 12 different *p* value thresholds were inserted as fixed effects in the models in addition to wave, gender, age, ancestry PCs. Similarly, intercepts for respondents and by-subject random slopes for time were entered as random effects.

In line with similar longitudinal studies investigating associations between substance use and depressive symptoms, linear mixed-effects models were used for statistical analyses (Anand, Paquette, Bartuska, & Daughters, 2019; Vermeulen et al., 2019). All analyses were performed in R (version 3.6.0) using the lme4 package. Outcomes of the variables of interest were presented as estimates (standard errors) and the corresponding intercept of the complete model. Models were fitted using restricted maximum likelihood (REML). Calculation of *p* values was done according to the Satterthwaite approach for all analyses with the pbkrtest package, which has the lowest type I error rates together with the Kenward-Roger approach for REML-fitted mixed-effects models (Halekoh & Højsgaard, 2015; Luke, 2017). The significance threshold was set at *p* < 0.05.

## Results

### Descriptive statistics

Data regarding smoking status were available for 6503 (97.8% of the total participants at this time point) individuals at baseline, and 5303 (100%) and 4618 (100%) individuals at the last two waves. In total, 1988 (30.6%) participants out of 6503 smoked at baseline, 1409 (26.6%) out of 5303 participants at the first follow-up assessment and 1076 (23.3%) out of 4618 participants at the second follow-up assessment (online Supplementary Table S2). In the three follow-up periods, respectively, 5443 out of 5763 (94.4%), 4182 out of 4209 (99.4%) and 3627 out of 3646 (99.5%) respondents used alcohol (online Supplementary Table S3). Cross-sectional baseline comparisons revealed that smoking and alcohol use were independent of each other. Additionally, baseline comparisons showed that the mean MHI-3 score was 17 for non-smoking and 16 for smoking people, indicating more depressive symptoms in smoking participants multi-cross-sectionally (*p* < 0.001; online Supplementary Table S2). At baseline, the mean MHI-3 score was 16 for both non-alcohol using and alcohol using people (*p* = 0.208; online Supplementary Table S3). Genetic data were available for 3104 participants of whom 1362 were male and 1742 female.

### Multi-cross-sectional results

Multi-cross-sectional linear mixed-model analysis showed that smoking was positively associated with depressive symptoms [estimate −0.36, standard error (s.e.) 0.04, *p* value <0.001; Table 1]. Furthermore, we found a positive association between the mean number of cigarettes per day and depressive symptoms (6–10 cigarettes: estimate −0.22, s.e. 0.07, *p* value 0.001; 11–20 cigarettes: estimate −0.48, s.e. 0.06, *p* value <0.001; >20 cigarettes: estimate −1.00, s.e. 0.09, *p* value <0.01; Table 2). No associations were found between alcohol use as a binary trait and depressive symptoms as well as between heavy drinking (10.5–84 drinks per week) and depressive symptoms (online Supplementary Tables S6 and S7). However, light to moderate drinking (1.5–10.5 drinks per week) was associated with less depressive symptoms (estimate 0.10, s.e. 0.047, *p* value 0.033; estimate 0.12, s.e. 0.048, *p* value 0.011, respectively; Table 3). Sensitivity analyses using the SF-36 outcome and correcting for additional covariates in all linear models confirmed our results, except that alcohol use as a binary trait was significantly negatively associated with depressive symptoms on the SF-36 and that light drinking (1.5–3.5 drinks per week) was not significantly associated with depressive symptoms on the MHI-3 (online Supplementary Tables S4–S7, S15, S17). Thus, across models, both smoking and smoking quantity were robustly associated with more depressive symptoms, while for alcohol, moderate drinking was robustly associated with less depressive symptoms.

**Table 1.** Multi-cross-sectional associations between smoking/non-smoking and symptoms of depression using the MHI-3 score (corrected for age and gender)

Smoking	Participants (*n* = 6646)		
Effects	Estimate	Std. Error	*p*
MHI-3 (3–18 points)			
**Intercept**	**16.113**	**0.038**	**<0.001**
**Smoking**	**−0.360**	**0.042**	**<0.001**
**3-year follow-up**	**−0.432**	**0.029**	**<0.001**
**6-year follow-up**	**−0.337**	**0.032**	**<0.001**

Fixed effects in the models were smoking, age, gender and time. Random effects were by-subject random slopes for time and intercepts for respondents. Data are estimates and *p* values were calculated using the Satterthwaite approach. Significant results (*p* value < 0.05) are provided in bold.

**Table 2.** Multi-cross-sectional associations between mean number of cigarettes per day and symptoms of depression using the MHI-3 score (corrected for age and gender)

	Participants (*n* = 6646)		
Effects	Estimate	Std. Error	*p*
	**MHI-3 (3–18 points)**		
**Intercept**	**16.130**	**0.038**	**<0.001**
<1 per week	−0.124	0.087	0.156
1–5 per day	−0.136	0.077	0.079
**6–10 per day**	**−0.223**	**0.067**	**0.001**
**11–20 per day**	**−0.476**	**0.059**	**<0.001**
**>20 per day**	**−1.003**	**0.090**	**<0.001**
**3-year follow-up**	**−0.439**	**0.029**	**<0.001**
**6-year follow-up**	**−0.348**	**0.032**	**<0.001**

Fixed effects in the models were cigarettes per day, age, gender and time. Random effects were by-subject random slopes for time and intercepts for respondents. Data are estimates and *p* values were calculated using the Satterthwaite approach. Significant results (*p* value < 0.05) are provided in bold.

**Table 3.** Multi-cross-sectional associations between mean number of drinks per week and symptoms of depression using the MHI-3 score (corrected for age and gender)

Alcohol drinks per week	Participants (*n* = 6646)		
Effects	Estimate	Std. Error	*p*
MHI-3 (3–18 points)			
**Intercept**	**16.013**	**0.048**	**<0.001**
**Drinks (1.5–3.5)**	**0.099**	**0.047**	**0.033**
**Drinks (3.5–10.5)**	**0.122**	**0.048**	**0.011**
Drinks (10.5–84.0)	−0.058	0.052	0.268
**3-year follow-up**	**−0.411**	**0.031**	**<0.001**
**6-year follow-up**	**−0.316**	**0.034**	**<0.001**

Fixed effects in the models were drinks per week, age, gender and time. Random effects were by-subject random slopes for time and intercepts for respondents. Data are estimates and *p* values were calculated using the Satterthwaite approach. Significant results (*p* value < 0.05) are provided in bold.

### Longitudinal analysis

In total, 978 (18.4%) participants of the total sample changed their smoking behaviour between baseline and 3-year follow-up [increase in the number of cigarettes in *n* = 384 (7.2%); decrease in the number of cigarettes in *n* = 594 (11.2%) participants] and 760 (16.5%) individuals between 3 and 6-year follow-up [increase *n* = 317 (6.9%); decrease *n* = 443 (9.6%) participants]. In total, 1783 (42.4%) respondents changed their alcohol behaviour between baseline and 3-year follow-up [increase in the number of drinks per week in *n* = 861 (20.5%); decrease in the number of drinks per week in *n* = 922 (21.9%) participants], and 1334 (36.6%) between 3 and 6-year follow-up [increase in *n* = 598 (16.4%); decrease in *n* = 736 (20.2%) participants]. Additional demographics on changes in independent variables are shown in online Supplementary Table S1.

Longitudinal analysis showed that a reduction of five levels in the number of cigarettes per day was associated with less depressive symptoms (estimate 0.76, s.e. 0.31, *p* value 0.015; Table 4). Smoking initiation and smoking cessation were not significantly associated with change in depressive symptoms (online Supplementary Table S8). Again, sensitivity analyses confirmed our findings (online Supplementary Table S18–S20).

**Table 4.** Longitudinal associations between change in cigarettes per day (per level) and symptoms of depression using the MHI-3 score (corrected for age and gender)

	Participants (*n* = 6646)		
Effects	Estimate	Std. Error	*p*
**MHI-3 (3–18 points)**			
**Intercept**	**−0.440**	**0.035**	<**0.001**
Increase 1 level	−0.005	0.087	0.958
Increase 2 levels	−0.219	0.179	0.221
Increase 3 levels	0.001	0.231	0.995
Increase 4 levels	0.014	0.261	0.958
Increase 5 levels	−0.842	0.592	0.156
Decrease 1 level	0.134	0.078	0.087
Decrease 2 levels	0.024	0.143	0.869
Decrease 3 levels	0.013	0.177	0.940
Decrease 4 levels	−0.293	0.154	0.058
**Decrease 5 levels**	**0.756**	**0.312**	**0.015**
**6-year follow-up**	**0.516**	**0.049**	<**0.01**

Fixed effects in the models were change in cigarettes per day, age, gender and time. Random effects were by-subject random slopes for time and intercepts for respondents. Data are estimates and *p* values were calculated using the Satterthwaite approach. Significant results (*p* value < 0.05) are provided in bold.

Alcohol cessation was associated with less depressive symptoms over time (estimate 0.63, standard error 0.29, *p* value 0.028; Table 5). Additionally, a decrease of two categories of drinks per week was associated with less depressive symptoms (estimate 0.36, standard error 0.11, *p* value 0.001; Table 6), which was confirmed in sensitivity analyses using additional covariates for a decrease of two and three categories of drinks per week (online Supplementary Table S23).

**Table 5.** Longitudinal associations between change in alcohol use and symptoms of depression using the MHI-3 score (corrected for age and gender)

	Participants (*n* = 6646)		
Effects	Estimate	Std. Error	*p*
**MHI-3 (3–18 points)**			
**Intercept**	**−0.421**	**0.036**	<**0.001**
**Quit alcohol use**	**0.630**	**0.286**	**0.028**
Start alcohol use	−0.020	0.217	0.926
**6-year follow-up**	**0.498**	**0.053**	<**0.001**

Fixed effects in the models were change in alcohol use, age, gender and time. Random effects were by-subject random slopes for time and intercepts for respondents. Data are estimates and *p* values were calculated using the Satterthwaite approach. Significant results (*p* value < 0.05) are provided in bold.

**Table 6.** Longitudinal associations between change in drinks per week (per level) and symptoms of depression using the MHI-3 score (corrected for age and gender)

	Participants (*n* = 6646)		
Effects	Estimate	Std. Error	*p*
**MHI-3 (3–18 points)**			
**Intercept**	**−0.437**	**0.040**	**<0.001**
Increase 1 level	−0.014	0.057	0.805
Increase 2 levels	−0.198	0.121	0.100
Increase 3 levels	0.160	0.301	0.594
Decrease 1 level	0.030	0.055	0.589
**Decrease 2 levels**	**0.358**	**0.110**	**0.001**
Decrease 3 levels	0.438	0.233	0.060
**6-year follow-up**	**0.507**	**0.054**	**<0.001**

Fixed effects in the models were change in drinks per week, age, gender and time. Random effects were by-subject random slopes for time and intercepts for respondents. Data are estimates and *p* values were calculated using the Satterthwaite approach. Significant results (*p* value < 0.05) are provided in bold.

### Validation analyses

All multi-cross-sectional and longitudinal results were similar for validation analyses using different thresholds for drinks per week for women and men (online Supplementary Tables S24–S37). Notably, we found additional significant results strengthening the abovementioned findings. For men, heavier drinking (21–84 drinks per week) was associated with more depressive symptoms multi-cross-sectionally (estimate −0.28, standard error 0.107, *p* value 0.010; online Supplementary Table S29). Decreasing drinks per week (binary) were associated with less depressive symptoms longitudinally for women and men (estimate 0.24, standard error 0.09, *p* value 0.012; online Supplementary Table S30, and estimate 0.23, standard error 0.09, *p* value 0.016; online Supplementary Table S27, respectively).

### Genetic risk score analyses

Directions of the effect of our PRS analyses of cigarettes per day and drinks per week were consistent with the results of multi-cross-sectional analysis (online Supplementary Tables S38–S39). Moreover, in line with our phenotypic associations, PRS for cigarettes per day were significantly associated with increased depressive symptoms using the SF-36 (P_T_ 5 × 10^−3^, *p* = 0.009; P_T_ 0.05, *p* = 0.017; P_T_ 0.1, *p* = 0.022; P_T_ 0.3, *p* = 0.034; P_T_ 0.4, *p* = 0.038; P_T_ 0.5, *p* = 0.031; online Supplementary Table S38).

## Discussion

In this prospective, longitudinal cohort study in the general population, smoking and number of cigarettes smoked per day were positively associated with depressive symptoms multi-cross-sectionally, whereas moderate drinking was associated with less depressive symptoms. Alcohol cessation and substantial reductions in both cigarette and alcohol consumption were associated with decreases in depressive symptoms. Extensive sensitivity, validation and PRS analyses corroborated the results.

Here, in accordance with previous literature, we found a higher prevalence of depressive symptoms in smoking participants compared to non-smoking participants cross-sectionally (Jamal et al., 2012; Lechner et al., 2019; Stepankova et al., 2017; Taylor et al., 2014). In contrast with several preceding observational studies and a meta-analysis, we found no association between smoking cessation and depressive symptoms over time (Stepankova et al., 2017; Taylor et al., 2014). Nevertheless, in line with Taylor et al., a decrease in the number of cigarettes was associated with less depressive symptoms. The latter may indicate that whenever an individual reduces the number of cigarettes smoked per day, effects on the severity of depressive symptoms may be expected. One possible explanation for the non-significant longitudinal results regarding smoking initiation, smoking cessation, alcohol initiation and change in depressive symptoms was the relatively low number of individuals changing binary smoking or alcohol behaviour, resulting in a lack of power.

Our finding that moderate alcohol use was associated with less depressive symptoms in the general population is in line with a recent study (Gémes et al., 2019). In contrast to Gémes et al., however, here heavy drinking was not associated with increases in depressive symptoms, which might be explained by different sample sizes. In the study of Gémes et al., 581 participants were categorized as hazardous drinkers of whom only 55 individuals drank >14 drinks per week, whereas in the current study, 1468 participants drank >10.5 drinks per week (online Supplementary Table S3). However, our validation analyses showed that heavier drinking in men is associated with more depressive symptoms. In a previous study, Lechner et al. reported no effect of alcohol intake reductions on depressive symptoms (Lechner et al., 2019). Nonetheless, we show that alcohol cessation or a substantial decrease in drinks (i.e. two or three categories) is associated with less depressive symptoms over time. The low number of participants (i.e. 150 heavy-drinking smokers) in the study of Lechner et al. may have restricted the power to detect this effect. Similarly to Haynes et al., heavier drinking was not associated with depressive symptoms but alcohol cessation was associated with a decrease in depressive symptoms (Haynes et al., 2005).

Several hypotheses regarding the association between smoking, alcohol use and depressive symptoms have been described. A frequently mentioned hypothesis is the self-medication theory of smoking and alcohol to treat symptoms of depression (Khantzian, 1997; O'Neil et al., 2011). The findings of the current observational, longitudinal study lend no support to the self-medication theory of nicotine and alcohol alleviating feelings of depression in the general population. According to the self-medication theory, smoking and alcohol use initiation and cessation should be negatively and positively associated with symptoms of depression, respectively. However, our observations that smoking was associated with more frequent symptoms of depression multi-cross-sectionally and that alcohol cessation and decreases in cigarettes and drinks per week were associated with less depressive symptoms, argue against the self-medication theory for both smoking and alcohol use. Additionally, conversely to what the self-medication theory assumes, neither smoking cessation, smoking initiation nor alcohol initiation was significantly associated with depressive symptoms. Furthermore, the misattribution hypothesis states that people misinterpret the relief of smoking and drinking withdrawal symptoms as the relief of depressive symptoms and explains why smoking or alcohol cessation leads to less depressive symptoms (McKenzie et al., 2010; Wootton et al., 2020). In support of this hypothesis, the results of this study indicated that alcohol cessation was associated with decreased depressive symptoms.

Shared genetic vulnerability, environmental interactions or direct causality has also been put forward as hypotheses to explain the association between smoking, alcohol use and depressive symptoms (Khantzian, 1997; O'Neil et al., 2011). However, findings in the literature on the temporal relationship between alcohol use, smoking and symptoms of depression are contradictory. Some prior studies suggest that depressive symptoms lead to substance use, while several studies conversely describe that SUD lead to depression or report bidirectional associations (Fergusson, Goodwin, & Horwood, 2003; Gémes et al., 2019; Grant & Harford, 1995; Hasin et al., 2005; Kuo et al., 2006; O'Neil et al., 2011; Polimanti et al., 2019; Wootton et al., 2020). A recent two-sample bi-directional Mendelian Randomisation (MR) study (Wootton et al., 2020) indicated that smoking is a causal risk factor for a diagnosis of depression, whereas previous MR studies found no evidence for a causal relation (Bjørngaard et al., 2013; Taylor et al., 2014; Wium-Andersen, Orsted, & Nordestgaard, 2015; Wootton et al., 2020). Similarly, several prospective studies report that alcohol abstinence is associated with reduced depressive symptoms while other studies found no effect on the course of symptoms of depression following alcohol abstinence or any level of intake-reduction (Brown et al., 1995; Brown & Schuckit, 1988; Lechner et al., 2019; Liappas et al., 2002).

Strengths of this study are the sample size, the prospective design, the range of sensitivity and validation analyses, and the longitudinal follow-up of three quantitative measures of smoking behaviour, alcohol use, depressive symptoms and genetic vulnerability to these traits over a 6-year period. Multiple covariates were added to the models, decreasing the risk of confounding. Naturally, due to the observational design, there was some loss to follow-up and some residual confounding and reverse causation may have occurred. Therefore, no definite conclusions regarding causality may be made. Another limitation was the lack of continuous information and data assessments beyond the past 4 weeks for smoking behaviour and 12 months for alcohol use, precluding conclusions about more acute or longer-term associations. Furthermore, misattribution and self-medication may have a complicated measurement of depressive symptoms (Bolton et al., 2009; Breslau et al., 2004; Grant & Harford, 1995; Khantzian, 1997; Kuo et al., 2006; McKenzie et al., 2010; Wootton et al., 2020). Lastly, the power for PRS analyses was limited.

In summary, the findings of this prospective, 6-year cohort study suggest that smoking and alcohol use are not associated with reduced depressive symptoms over time. Our multi-cross-sectional results provide evidence that smoking and the number of cigarettes smoked per day are positively associated with depressive symptoms. While moderate drinkers experienced fewer depressive symptoms cross-sectionally, heavier drinking in men was associated with more depressive symptoms in our validation analyses, which is in line with previous literature (Gémes et al., 2019). In addition, alcohol cessation or decrease in drinks per week was associated with reduced depressive symptoms longitudinally, supporting the results of several prospective studies (Brown et al., 1995; Brown & Schuckit, 1988; Liappas et al., 2002). Future studies taking genetic risks into account are needed to examine the quantitative, temporal relationship between smoking, alcohol use and depressive symptoms to confirm the results of this study and to explore the underlying mechanisms. As ethical considerations render randomization unlikely for smoking and alcohol behaviours and in light of the observational design of the current study, future MR and (genomic) Structural Equation Modelling studies focused on changes in the variables we investigated may shed light on causality (Grotzinger et al., 2019). This study addressed depressive symptoms and alcohol use instead of diagnosis of depression or alcohol use disorder. As such, our results contribute to the elucidation of the associations between smoking, alcohol use and depressive symptoms in the general population and, therefore, inform the development of future effective prevention strategies for public mental health and for clinical psychiatry, where not only depression as a categorical diagnosis but also depressive symptoms are frequently observed.

## Data Availability

Data are available upon reasonable request.

## Supplementary material

For supplementary material accompanying this paper visit https://doi.org/10.1017/S0033291721002968. click here to view supplementary material

## References

[ref1] American Psychiatric Association Task Force on DSM-IV. (2000). *Diagnostic and statistical manual of mental disorders: DSM-IV-TR*. Retrieved May 28, 2019, from https://olin.tind.io/record/123204/.

[ref2] Anand, D., Paquette, C., Bartuska, A., & Daughters, S. (2019). Substance type moderates the longitudinal association between depression and substance use from pre-treatment through a 1-year follow-up. Drug and Alcohol Dependence, 197, 87–94. doi: 10.1016/j.drugalcdep.2019.01.002. 30784954 PMC8805280

[ref3] Anda, R. F., Williamson, D. F., Escobedo, L. G., Mast, E. E., Giovino, G. A., & Remington, P. L. (1990). Depression and the dynamics of smoking. JAMA, 264(12), 1541. 10.1001/jama.1990.03450120053028. 2395193

[ref4] Anthony, J. C., Warner, L. A., & Kessler, R. C. (1994). Comparative epidemiology of dependence on tobacco, alcohol, controlled substances, and inhalants: Basic findings from the National Comorbidity Survey. Experimental and Clinical Psychopharmacology, 2(3), 244–268. 10.1037/1064-1297.2.3.244.

[ref5] Bjørngaard, J. H., Gunnell, D., Elvestad, M. B., Smith, G. D., Skorpen, F., Krokan, H., … Romundstad, P. (2013). The causal role of smoking in anxiety and depression: A Mendelian randomization analysis of the HUNT study. Psychological Medicine, 43(4), 711–719. 10.1017/S0033291712001274. 22687325

[ref6] Boden, J. M., & Fergusson, D. M. (2011). Alcohol and depression. Addiction, 106(5), 906–914. 10.1111/j.1360-0443.2010.03351.x. 21382111

[ref7] Boden, J. M., Fergusson, D. M., & Horwood, L. J. (2010). Cigarette smoking and depression: Tests of causal linkages using a longitudinal birth cohort. British Journal of Psychiatry, 196(6), 440–446. 10.1192/bjp.bp.109.065912. 20513853

[ref8] Bolton, J. M., Robinson, J., & Sareen, J. (2009). Self-medication of mood disorders with alcohol and drugs in the National Epidemiologic Survey on Alcohol and Related Conditions. Journal of Affective Disorders, 115(3), 367–375. 10.1016/J.JAD.2008.10.003. 19004504

[ref9] Bos, E. H., ten Have, M., van Dorsselaer, S., Jeronimus, B. F., de Graaf, R., & de Jonge, P. (2018). Functioning before and after a major depressive episode: Pre-existing vulnerability or scar? A prospective three-wave population-based study. Psychological Medicine, 48(13), 2264–2272. 10.1017/S0033291717003798. 29331152

[ref10] Breslau, N., Novak, S. P., & Kessler, R. C. (2004). Daily smoking and the subsequent onset of psychiatric disorders. Psychological Medicine, 34(2), 323–333. 14982138 10.1017/s0033291703008869

[ref11] Brown, S. A., Inaba, R. K., Gillin, J. C., Schuckit, M. A., Stewart, M. A., & Irwin, M. R. (1995). Alcoholism and affective disorder: Clinical course of depressive symptoms. American Journal of Psychiatry, 152(1), 45–52. 7802119 10.1176/ajp.152.1.45

[ref12] Brown, R. A., Lewinsohn, P. M., Seeley, J. R., & Wagner, E. F. (1996). Cigarette smoking, major depression, and other psychiatric disorders among adolescents. Journal of the American Academy of Child & Adolescent Psychiatry, 35(12), 1602–1610. 10.1097/00004583-199612000-00011. 8973066

[ref13] Brown, S. A., & Schuckit, M. A. (1988). Changes in depression among abstinent alcoholics*. Journal of Studies on Alcohol, 49(5), 412–417. 3216643 10.15288/jsa.1988.49.412

[ref14] Brugha, T. S., & Cragg, D. (1990). The list of threatening experiences: The reliability and validity of a brief life events questionnaire. Acta Psychiatrica Scandinavica, 82(1), 77–81. 10.1111/j.1600-0447.1990.tb01360.x. 2399824

[ref15] Cabello, M., Miret, M., Caballero, F. F., Chatterji, S., Naidoo, N., Kowal, P., … Ayuso-Mateos, J. L. (2017). The role of unhealthy lifestyles in the incidence and persistence of depression: A longitudinal general population study in four emerging countries. Globalization and Health, 13(1), 18. 10.1186/s12992-017-0237-5. 28320427 PMC5358047

[ref16] Chaiton, M., Cohen, J. E., Rehm, J., Abdulle, M., & O'loughlin, J. (2014). *Confounders or intermediate variables? Testing mechanisms for the relationship between depression and smoking in a longitudinal cohort study*. 10.1016/j.addbeh.2014.11.026. 25462665

[ref17] Choi, S. W., Mak, T. S.-H., & O'Reilly, P. F. (2020). Tutorial: A guide to performing polygenic risk score analyses. Nature Protocols, 15(9), 2759–2772. 10.1038/s41596-020-0353-1. 32709988 PMC7612115

[ref18] Cuijpers, P., Smit, F., ten Have, M., & de Graaf, R. (2007). Smoking is associated with first-ever incidence of mental disorders: A prospective population-based study. Addiction, 102(8), 1303–1309. 10.1111/j.1360-0443.2007.01885.x. 17624980

[ref19] Davis, L. L., Frazier, E., Husain, M. M., Warden, D., Trivedi, M., Fava, M., … Rush, A. J. (2006). Substance use disorder comorbidity in major depressive disorder: A confirmatory analysis of the STAR*D cohort. American Journal on Addictions, 15(4), 278–285. 10.1080/10550490600754317. 16867922

[ref20] Degenhardt, L., Charlson, F., Ferrari, A., Santomauro, D., Erskine, H., Mantilla-Herrara, A., … Vos, T. (2018). The global burden of disease attributable to alcohol and drug use in 195 countries and territories, 1990–2016: A systematic analysis for the Global Burden of Disease Study 2016. The Lancet Psychiatry, 5(12), 987–1012. 10.1016/S2215-0366(18)30337-7. 30392731 PMC6251968

[ref21] De Graaf, R., Ten Have, M., & van Dorsselaer, S. (2010). The Netherlands Mental Health Survey and Incidence Study-2 (NEMESIS-2): design and methods. Int J Methods Psychiatr Res, 19(3), 125–141. Doi: 10.1002/mpr.317. 20641046 PMC6878518

[ref22] Des Jarlais, D. C., Lyles, C., & Crepaz, N., & the TREND Group (2004). Improving the reporting quality of nonrandomized evaluations of behavioral and public health interventions: The TREND statement. American Journal of Public Health, 94(3), 361–366. 10.2105/ajph.94.3.361. 14998794 PMC1448256

[ref23] De Staat van Volksgezondheid en Zorg. (2020). Alcoholgebruik: overmatige drinkers. Retrieved March 1, 2021, from https://www.staatvenz.nl/kerncijfers/alcoholgebruik-overmatige-drinkers.

[ref24] European Monitoring Centre for Drugs and Drug Addiction. (2006). *Annual Report* 2006*: The state of the drug problem in Europe. Luxembourg: Office for Official Publications of the European Communities*. Retrieved May 28, 2019, from https://www.emcdda.europa.eu/system/files/publications/924/ar2006-en_69466.pdf.

[ref25] European Monitoring Centre for Drugs and Drug Addiction. (2015). Comorbidity of substance use and mental disorders in Europe. Luxembourg: Office for Official Publications of the European Communities. Retrieved May 28, 2019, from https://www.emcdda.europa.eu/system/files/publications/1988/TDXD15019ENN.pdf.

[ref26] Ezzati, M., Lopez, A. D., Rodgers, A., Vander Hoorn, S., & Murray, C. J. (2002). Selected major risk factors and global and regional burden of disease. The Lancet, 360(9343), 1347–1360. 10.1016/S0140-6736(02)11403-6. 12423980

[ref27] Fergusson, D. M., Boden, J. M., & Horwood, L. J. (2009). Tests of causal links between alcohol abuse or dependence and major depression. Archives of General Psychiatry, 66(3), 260. 10.1001/archgenpsychiatry.2008.543. 19255375

[ref28] Fergusson, D. M., Goodwin, R. D., & Horwood, L. J. (2003). Major depression and cigarette smoking: Results of a 21-year longitudinal study. Psychological Medicine, 33(8), 1357–1367. 14672244 10.1017/s0033291703008596

[ref29] Flensborg-Madsen, T., Mortensen, E. L., Knop, J., Becker, U., Sher, L., & Grønbæk, M. (2009). Comorbidity and temporal ordering of alcohol use disorders and other psychiatric disorders: Results from a Danish register-based study. Comprehensive Psychiatry, 50(4), 307–314. 10.1016/j.comppsych.2008.09.003. 19486728

[ref30] Gémes, K., Forsell, Y., Janszky, I., László, K. D., Lundin, A., Ponce De Leon, A., … Moller, J. (2019). Moderate alcohol consumption and depression – a longitudinal population-based study in Sweden. Acta Psychiatrica Scandinavica, 139(6), 526–535. 10.1111/acps.13034. 30980542

[ref31] Gilman, S. E., & Abraham, H. D. (2001). A longitudinal study of the order of onset of alcohol dependence and major depression. Drug and Alcohol Dependence, 63(3), 277–286. 10.1016/S0376-8716(00)00216-7. 11418232

[ref32] Glassman, A. H., Helzer, J. E., Covey, L. S., Cottler, L. B., Stetner, F., Tipp, J. E., & Johnson, J. (1990). Smoking, smoking cessation, and major depression. JAMA, 264(12), 1546. 10.1001/jama.1990.03450120058029. 2395194

[ref33] Grant, B. F., & Harford, T. C. (1995). Comorbidity between DSM-IV alcohol use disorders and major depression: Results of a national survey. Drug and Alcohol Dependence, 39(3), 197–206. 10.1016/0376-8716(95)01160-4. 8556968

[ref34] Groenman, A. P., Janssen, T. W. P., & Oosterlaan, J. (2017). Childhood psychiatric disorders as risk factor for subsequent substance abuse: A meta-analysis. Journal of the American Academy of Child and Adolescent Psychiatry, 56(7), 556–569. 10.1016/j.jaac.2017.05.004. 28647007

[ref35] Grotzinger, A. D., Rhemtulla, M., de Vlaming, R., Ritchie, S. J., Mallard, T. T., Hill, W. D., … Tucker-Drob, E. M. (2019). Genomic structural equation modelling provides insights into the multivariate genetic architecture of complex traits. Nature Human Behaviour, 3(5), 513–525. 10.1038/s41562-019-0566-x. PMC6520146 30962613

[ref36] Guloksuz, S., Pries, L., Have, M., Graaf, R., Dorsselaer, S., Klingenberg, B., … Os, J. (2020). Association of preceding psychosis risk states and non-psychotic mental disorders with incidence of clinical psychosis in the general population: A prospective study in the NEMESIS-2 cohort. World Psychiatry, 19(2), 199–205. 10.1002/wps.20755. 32394548 PMC7215054

[ref37] Halekoh, U., & Højsgaard, S. (2015). A Kenward-Roger approximation and parametric bootstrap methods for tests in linear mixed models – The R package pbkrtest. Journal of Statistical Software, 59, 9. 10.18637/jss.v059.i09.

[ref38] Hasin, D. S., Goodwin, R. D., Stinson, F. S., & Grant, B. F. (2005). Epidemiology of major depressive disorder. Archives of General Psychiatry, 62(10), 1097. 10.1001/archpsyc.62.10.1097. 16203955

[ref39] Haynes, J. C., Farrell, M., Singleton, N., Meltzer, H., Araya, R., Lewis, G., & Wiles, N. J. (2005). Alcohol consumption as a risk factor for anxiety and depression. British Journal of Psychiatry. Cambridge University Press, 187(6), 544–551. 10.1192/bjp.187.6.544. 16319407

[ref40] Hooshmand, S., Willoughby, T., & Good, M. (2012). Does the direction of effects in the association between depressive symptoms and health-risk behaviors differ by behavior? A longitudinal study across the high school years. The Journal of Adolescent Health: Official Publication of the Society for Adolescent Medicine, 50(2), 140–147. 10.1016/j.jadohealth.2011.05.016. 22265109

[ref41] Jamal, M., Willem Van der Does, A. J., Cuijpers, P., & Penninx, B. W. J. H. (2012). Association of smoking and nicotine dependence with severity and course of symptoms in patients with depressive or anxiety disorder. Drug and Alcohol Dependence, 126(1–2), 138–146. 10.1016/J.DRUGALCDEP.2012.05.001. 22633368

[ref42] Kaplow, J. B., Curran, P. J., Angold, A., & Costello, E. J. (2001). The prospective relation between dimensions of anxiety and the initiation of adolescent alcohol use. Journal of Clinical Child Psychology, 30(3), 316–326. 10.1207/S15374424JCCP3003_4. 11501249

[ref43] Kessler, R. C., Chiu, W. T., Demler, O., Walters, E. E., & Walters, E. E. (2005). Prevalence, severity, and comorbidity of 12-month DSM-IV disorders in the National Comorbidity Survey Replication. Archives of General Psychiatry, 62(6), 617. 10.1001/archpsyc.62.6.617. 15939839 PMC2847357

[ref44] Kessler, R. C., Crum, R. M., Warner, L. A., Nelson, C. B., Schulenberg, J., & Anthony, J. C. (1997). Lifetime co-occurrence of DSM-III-R alcohol abuse and dependence with other psychiatric disorders in the National Comorbidity Survey. Archives of General Psychiatry, 54(4), 313. 10.1001/archpsyc.1997.01830160031005. 9107147

[ref45] Khantzian, E. J. (1997). The self-medication hypothesis of substance use disorders: A reconsideration and recent applications. Harvard Review of Psychiatry, 4(5), 231–244. 10.3109/10673229709030550. 9385000

[ref46] Kotov, R., Guey, L. T., Bromet, E. J., & Schwartz, J. E. (2010). Smoking in schizophrenia: Diagnostic specificity, symptom correlates, and illness severity. Schizophrenia Bulletin, 36(1), 173–181. 10.1093/schbul/sbn066. 18562340 PMC2800136

[ref47] Kuo, P.-H., Gardner, C. O., Kendler, K. S., & Prescott, C. A. (2006). The temporal relationship of the onsets of alcohol dependence and major depression: Using a genetically informative study design. Psychological Medicine, 36(8), 1153–1162. 10.1017/S0033291706007860. 16734951

[ref48] Lechner, W. V., Sidhu, N. K., Cioe, P. A., & Kahler, C. W. (2019). Effects of time-varying changes in tobacco and alcohol use on depressive symptoms following pharmaco-behavioral treatment for smoking and heavy drinking. Drug and Alcohol Dependence, 194, 173–177. 10.1016/J.DRUGALCDEP.2018.09.030. 30445275 PMC7364819

[ref49] Liappas, J., Paparrigopoulos, T., Tzavellas, E., & Christodoulou, G. (2002). *Impact of alcohol detoxification on anxiety and depressive symptoms*. Retrieved from www.elsevier.com/locate/drugalcdep. 10.1016/s0376-8716(02)00195-3 12234651

[ref50] Luke, S. G. (2017). Evaluating significance in linear mixed-effects models in R. Behavior Research Methods, 49(4), 1494–1502. 10.3758/s13428-016-0809-y. 27620283

[ref51] McHorney, C. A., Ware, J. E., Rachel Lu, J. F., & Sherbourne, C. D. (1994). The MOS 36-ltem Short-Form Health Survey (SF-36): III. Tests of data quality, scaling assumptions, and reliability across diverse patient groups. Medical Care, 32(1), 40–66. 10.1097/00005650-199401000-00004. 8277801

[ref52] McKenzie, M., Olsson, C. A., Jorm, A. F., Romaniuk, H., & Patton, G. C. (2010). Association of adolescent symptoms of depression and anxiety with daily smoking and nicotine dependence in young adulthood: Findings from a 10-year longitudinal study. Addiction, 105(9), 1652–1659. 10.1111/j.1360-0443.2010.03002.x. 20707783

[ref53] O'Neil, K. A., Conner, B. T., & Kendall, P. C. (2011). Internalizing disorders and substance use disorders in youth: Comorbidity, risk, temporal order, and implications for intervention. Clinical Psychology Review, 31(1), 104–112. 10.1016/J.CPR.2010.08.002. 20817371

[ref54] Pérez-Stable, E. J., Marín, G., Marín, B. V., & Katz, M. H. (1990). Depressive symptoms and cigarette smoking among Latinos in San Francisco. American Journal of Public Health, 80(12), 1500–1502. 2240340 10.2105/ajph.80.12.1500 PMC1405110

[ref55] Polimanti, R., Peterson, R. E., Ong, J.-S., MacGregor, S., Edwards, A. C., Clarke, T.-K., … Derks, E. M. (2019). Evidence of causal effect of major depression on alcohol dependence: Findings from the Psychiatric Genomics Consortium. Psychol Med., 49(7), 1218–1226. 10.1017/S0033291719000667. 30929657 PMC6565601

[ref56] Pomerleau, C. S., Zucker, A. N., & Stewart, A. J. (2003). Patterns of depressive symptomatology in women smokers, ex-smokers, and never-smokers. Addictive Behaviors, 28(3), 575–582. Retrieved from http://www.ncbi.nlm.nih.gov/pubmed/12628628. 12628628 10.1016/s0306-4603(01)00257-x

[ref57] Pries, L.-K., Guloksuz, S., Ten Have, M., de Graaf, R., van Dorsselaer, S., Gunther, N., … van Os, J. (2018). Evidence that environmental and familial risks for psychosis additively impact a multidimensional subthreshold psychosis syndrome. Schizophrenia Bulletin, 44(4), 710–719. 10.1093/schbul/sby051. 29701807 PMC6007403

[ref58] Stepankova, L., Kralikova, E., Zvolska, K., Pankova, A., Ovesna, P., Blaha, M., & Brose, L. S. (2017). Depression and smoking cessation: Evidence from a smoking cessation clinic with 1-year follow-up. Annals of Behavioral Medicine: A Publication of the Society of Behavioral Medicine, 51(3), 454–463. 10.1007/s12160-016-9869-6. 28035641 PMC5440483

[ref59] Sullivan, L. E., Fiellin, D. A., & O'Connor, P. G. (2005). The prevalence and impact of alcohol problems in major depression: A systematic review. The American Journal of Medicine, 118(4), 330–341. 10.1016/j.amjmed.2005.01.007. 15808128

[ref60] Swendsen, J. D., Merikangas, K. R., Canino, G. J., Kessler, R. C., Rubio-Stipec, M., & Angst, J. (1998). The comorbidity of alcoholism with anxiety and depressive disorders in four geographic communities. Comprehensive Psychiatry, 39(4), 176–184. 10.1016/S0010-440X(98)90058-X. 9675501

[ref61] Taylor, G., McNeill, A., Girling, A., Farley, A., Lindson-Hawley, N., & Aveyard, P. (2014). Change in mental health after smoking cessation: Systematic review and meta-analysis. BMJ *(*Clinical Research Ed.*)*, 348, g1151. 10.1136/bmj.g1151. PMC3923980 24524926

[ref62] Troisi, A., Pasini, A., Saracco, M., & Spalletta, G. (1998). Psychiatric symptoms in male cannabis users not using other illicit drugs. Addiction, 93(4), 487–492. 10.1046/j.1360-0443.1998.9344874.x. 9684387

[ref63] Tuithof, M., Ten Have, M., van den Brink, W., Vollebergh, W., & de Graaf, R. (2013). Predicting persistency of DSM-5 alcohol use disorder and examining drinking patterns of recently remitted individuals: A prospective general population study. Addiction *(*Abingdon, England*)*, 108(12), 2091–2099. 10.1111/add.12309. 23889861

[ref64] Vermeulen, J. M., Schirmbeck, F., Blankers, M., van Tricht, M., Bruggeman, R., van den Brink, W., … van Winkel, R. (2018). Association between smoking behavior and cognitive functioning in patients with psychosis, siblings, and healthy control subjects: Results from a prospective 6-year follow-up study. American Journal of Psychiatry, 175(11), 1121–1128. 10.1176/appi.ajp.2018.18010069. 30138044

[ref65] Vermeulen, J., Schirmbeck, F., Blankers, M., van Tricht, M., van den Brink, W., de Haan, L., … van Winkel, R. (2019). Smoking, symptoms, and quality of life in patients with psychosis, siblings, and healthy controls: A prospective, longitudinal cohort study. The Lancet Psychiatry, 6(1), 25–34. 10.1016/S2215-0366(18)30424-3. 30527763

[ref66] von Elm, E., Altman, D. G., Egger, M., Pocock, S. J., Gøtzsche, P. C., & Vandenbroucke, J. P. (2007). The Strengthening the Reporting of Observational Studies in Epidemiology (STROBE) statement: Guidelines for reporting observational studies. Annals of Internal Medicine, 147(8), 573. 10.7326/0003-4819-147-8-200710160-00010. 17938396

[ref67] Ware, J. E., & Sherbourne, C. D. (1992). *The MOS* 36*-Item Short-Form Health Survey* (*SF-*36) *I*. *Conceptual Framework and Item Selection* (Vol. 30). Retrieved from https://pdfs.semanticscholar.org/06cb/0076e310136d0ca8b56cc8585ec2bf43e029.pdf. 1593914

[ref68] Wilkinson, A. L., Halpern, C. T., & Herring, A. H. (2016). Directions of the relationship between substance use and depressive symptoms from adolescence to young adulthood. Addictive Behaviors, 60, 64–70. 10.1016/J.ADDBEH.2016.03.036. 27100470 PMC4884464

[ref69] Wium-Andersen, M. K., Orsted, D. D., & Nordestgaard, B. G. (2015). Tobacco smoking is causally associated with antipsychotic medication use and schizophrenia, but not with antidepressant medication use or depression. International Journal of Epidemiology, 44(2), 566–577. 10.1093/ije/dyv090. 26054357

[ref71] Wootton, R. E., Richmond, R.. C, Stuijfzand, B. G., Lawn, R. B., Sallis, H. M., Taylor, G. M. J., … Munafò, M. R. (2020). Evidence for causal effects of lifetime smoking on risk for depression and schizophrenia: a Mendelian randomisation study. Psychol Med, 50(14), 2435–2443. 10.1017/S0033291719002678. Epub 2019 Nov 6. 31689377 PMC7610182

[ref72] Yamazaki, S., Fukuhara, S., & Green, J. (2005). Usefulness of five-item and three-item Mental Health Inventories to screen for depressive symptoms in the general population of Japan. Health and Quality of Life Outcomes, 3(1), 48. 10.1186/1477-7525-3-48. 16083512 PMC1201161

