# Supplementary material for: Longitudinal associations between alcohol use, smoking, genetic risk scoring and symptoms of depression in the general population: a prospective 6-year cohort study
Source: Psychol Med. 2021 Aug 11;53(4):1409–17. doi: 10.1017/S0033291721002968 (PMC10009403; doi:10.1017/S0033291721002968)
Supplement: Supplementary file 1 [file S0033291721002968sup001.docx]

**Supplemental Material –**

**Longitudinal associations between alcohol use, smoking, genetic risk scoring, and symptoms of depression in the general population: a prospective six-year cohort study**

Nini de Boer^1^ MD, Jentien M. Vermeulen^2^ MD, PhD, Bochao Danae Lin^3^ PhD, Jim van Os^1,4,5^, Margreet ten Have PhD^6^, Ron de Graaf PhD^6^, Saskia van Dorsselaer MSc^6^, Maarten Bak MD^4,7^, PhD, Bart P.F. Rutten MD, PhD^4^, Albert Batalla^1^ MD, PhD, Sinan Guloksuz^4,8^ MD, PhD, Jurjen J Luykx ^1,3,9^ MD, PhD

^1^Department of Psychiatry, UMC Utrecht Brain Center, University Medical Center Utrecht, Utrecht University, Utrecht, The Netherlands

^2^Department of Psychiatry, Amsterdam UMC location AMC, University of Amsterdam, Amsterdam, the Netherlands

^3^Department of Translational Neuroscience, UMC Utrecht Brain Center, University Medical Center Utrecht, Utrecht University, Utrecht, the Netherlands

^4^Department of Psychiatry and Neuropsychology, School for Mental Health and Neuroscience, Maastricht University Medical Centre, Maastricht, the Netherlands

^5^ Department of Psychosis Studies, Institute of Psychiatry, Psychology & Neuroscience, King’s College London, London, United Kingdom

^6^ Department of Epidemiology, Netherlands Institute of Mental Health and Addiction, Utrecht, The Netherlands

^7^ FACT, Mondriaan Mental Health, Maastricht, the Netherlands

^8^ Department of Psychiatry, Yale University School of Medicine, New Haven, CT

^9^GGNet Mental Health, Apeldoorn, the Netherlands

**Appendix**

**Contents**

Additional information regarding dependent variables (SF-36 subscale score and MHI-3) p.2

Additional information regarding PRS analysis p.4

Supplementary results (supplementary tables S1 – S47) p.9

**Additional information regarding dependent variables**

The Short Form 36 Health Survey (SF-36) is a self-report questionnaire consisting of nine subscales each ranging from 0-100 points and symbolising poor to good functioning (McHorney, Ware, Rachel Lu, & Sherbourne, 1994). As sensitivity outcome, the scores of three subscales of the SF-36 (i.e. the mental health-scale or the Mental Health Inventory-5 (MHI-5), the vitality-scale and the emotional role functioning-scale) were combined into one scale (total 0-300 points), indicating the level of depressive symptoms in the past four weeks, similarly to Bos *et al.* (Bos et al., 2018). As primary outcome, three questions of the MHI-5 subscale were used, forming the Mental Health Inventory-3 (MHI-3).

*Subscales SF-36:*

*Scale 4 - Emotional role functioning*

During the past 4 weeks, have you had any of the following problems with your work or other regular daily activities as a result of any emotional problems (such as feeling depressed or anxious?

5a       Cut down the amount of time you spent on work or other activities

5b        Accomplished less than you would like

5c         Didn’t do work or other activities as carefully as usual

*Scale 5 - Mental Health Inventory – 5*

These questions are about how you feel and how things have been with you during the past 4 weeks. For each question, please give the one answer that comes closest to the way you have been feeling. How much of the time during the past 4 weeks..

9b Have you been a very nervous person?

9c       Have you felt so down in the dumps that nothing could cheer you up?

9d       Did you felt calm and peaceful?

9f        Have you felt downhearted and blue?

9h       Have you been a happy person?

*Scale 6 - Vitality*

These questions are about how you feel and how things have been with you during the past 4 weeks. For each question, please give the one answer that comes closest to the way you have been feeling. How much of the time during the past 4 weeks..

9a        Did you feel full of pep?

9e        Did you have a lot of energy?

9g       Did you feel worn out?

9i        Did you feel tired?

Primary outcome: subscale 5 question 9c, 9f, 9h (MHI-3)

Sensitivity outcome: subscale 4,5,6 (SF-36 subscale score)

**Additional information regarding PRS analysis**

For the purpose of genetic analyses, polygenic risk scores (PRS) were computed in all genotyped participants for three phenotypes -alcohol drinks per week, smoking initiation and cigarettes per day- for twelve p-value thresholds (0.5, 0.4, 0.3, 0.2, 0.1, 0.05, 5e-3, 5e-4, 5e-5, 5e-6, 5e-7, 5e-8). PRS estimate the genetic liability of an individual for specific traits or diseases (Choi *et al.* 2020). Summary statistics from the latest and largest genome-wide association studies (GWASs) were used for this.(Liu et al., 2019; Walters et al., 2018). Pre-imputation and post-imputation quality control was applied in PLINK v1.9 (Purcell et al., 2007). Insertions and deletions, ambiguous single nucleotide polymorphisms (SNPs), SNPs with a minor allele frequency < 0.01 and/or imputation quality R2 < 0.9, as well as SNPs located in complex-linkage disequilibrium (LD) regions and long-range LD regions were excluded (supplementary table S47). PRS generation was performed using PRSice (Euesden *et al.*, 2015) See below for further details regarding PRS analyses (quality control, imputation, and principle component analyses).

1. Genotyping procedures and quality control steps before imputation.

NEMESIS-2 samples were genotyped on the IPMCN chip (Institute of Psychological Medicine and Clinical Neurology, Cardiff University (588,628 genotyped common variants)) (European Network of National Networks studying Gene-Environment Interactions in Schizophrenia (EU-GEI), 2014). Quality control (QC) was done using PLINK v1.9 (Purcell et al., 2007) as follows. There were 3,861 samples matching with phenotypes. Single nucleotide polymorphisms (SNPs) and samples with call rates below 95% and 98%, respectively, were removed. A strict SNP QC only for subsequent sample QC steps was conducted. This involved a minor allele frequency (MAF) threshold > 10% and a Hardy-Weinberg equilibrium (HWE) P-value > 10-5, followed by linkage disequilibrium (LD) based SNP pruning (R2 < 0.5; supplementary table S33). This resulted in ~60K SNPs to assess sex errors (n=145), heterozygosity (F< 5xSD the standard deviation (SD), n= 73), and relatedness by pairwise identity by descent (IBD) values > 0.1 (n= 170). Genetic outliers (n=154) were identified by principal component analysis (PCA, see below). In total, 3,104 individuals passed these QC steps. After removing failing samples (n = 757), a regular SNP QC was performed (SNP call rate>95%, HWE p>1e-06, MAF> 0.16%; as the IPMCN chip contains many rare variants, half the SNPs would have been removed if we had applied MAF> 1%; therefore, we loosened MAF threshold to 0.16% =10/(2*sample size of 3,104)). Next, strand ambiguous SNPs and duplicate SNPs were removed, resulting in a total of 298,104 genotyped variants.

2. Imputation on Michigan server.

The QC-ed dataset was chunked by chromosome, and then converted into *.VCF files. The Michigan server was used for imputation with the following settings: reference panel as HRC R1.1 2016; phasing as Eagle v2.3; population as European; model as QC & imputation. The imputation resulted in 47,101,073 single nucleotide polymorphisms (SNPs). The general imputation quality is shown in Figure S1.

## **Figure S1. Correlation of SNPs MAF from NEMESIS-2 dataset with the reference MAF**


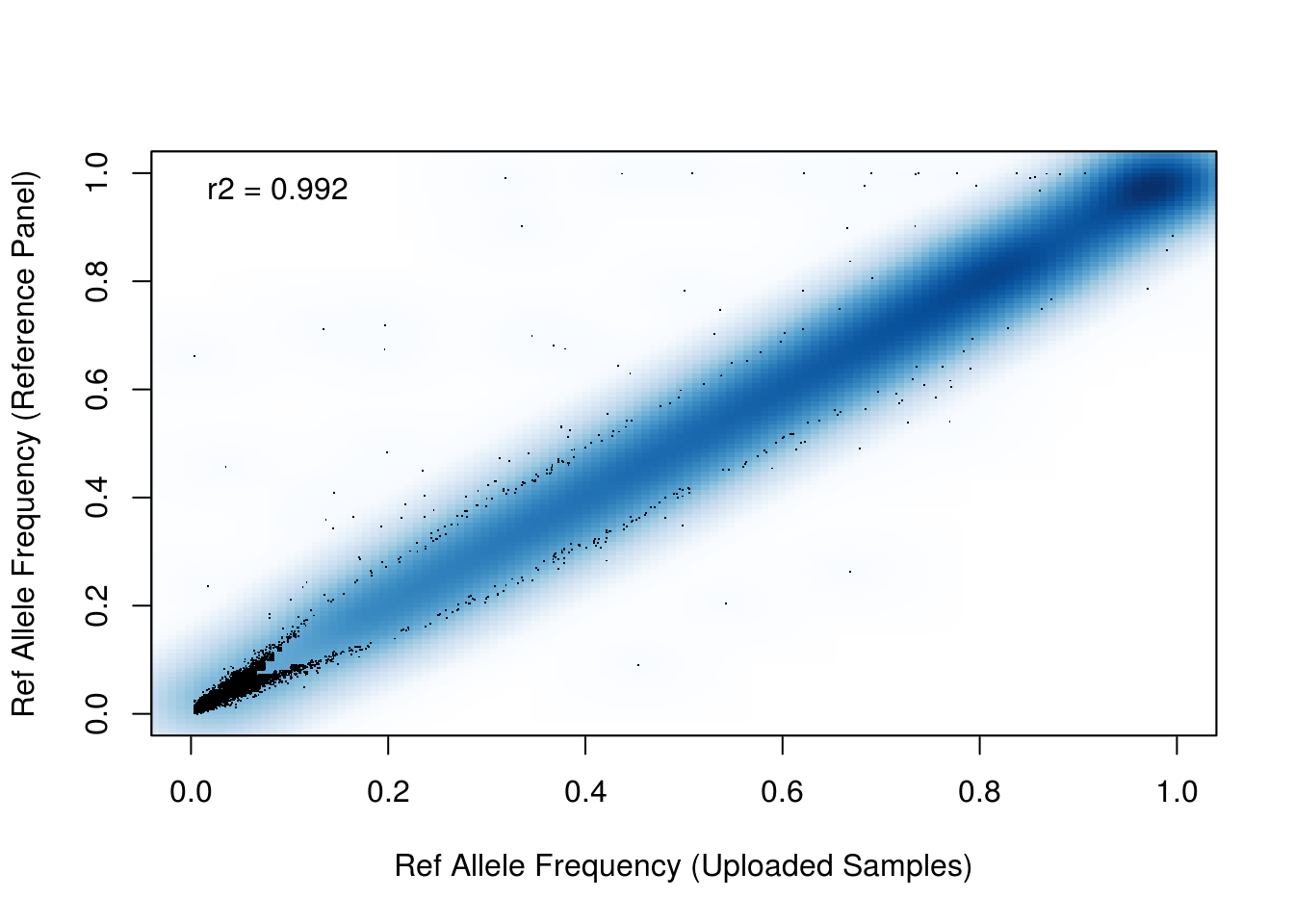


3. Quality control after imputation.

Poor quality SNPs were excluded: multi-allelic SNPs, SNPs with a minor allele frequency (MAF)<0.0016 or INFO<0.3, and strand ambiguous AT/CG SNPs. The VCF dosage files were converted into PLINK format dosage files. Finally, 10,356,437 SNPs and 3,104 individuals remained. For hard call (best guess) genotypes, an additional SNP QC (INFO>0.8 and HWE>10-6) was performed, resulting in 6,436,459 SNPs.

4. Principle components analyses (PCA).

The principal components (PCs) analyses within NEMESIS-2 samples, and NEMESIS-2 samples along with Hapmap3 (Wellcome Sanger Institute, 2021 ) populations were conducted by EIGENSTRAT (Price et al., 2006). A strict selection for SNPs with overlap with Hapmap3 SNPs was conducted: 1. MAF>0.05 and HWE>0.001; 2. Removal of 24 long LD regions; 3. LD pruning with an R2 of 0.5; which resulted in 40,732 best quality genotyped SNPs used to calculate genetic PCs (supplementary table S33). PCs were firstly calculated with HapMap3 population to exclude European ethnic outliers: exceeding 5 times the standard deviation of Utah residents with Northern and Western European ancestry from the CEPH collection (CEU) and Toscani in Italia (TSI) populations for the first 2 PCs. The first 2 PCs explained > 84.3% of the total. In total, 141 individuals were excluded from this dataset. Secondly, another PCA was conducted using the same SNPs (n=40,732) but calculated only in NEMESIS-2. The first 2 PCs explained >30% of the total. Another 13 individuals were considered as ethnic outliers by the first 2 PCs exceeding 5 times the standard deviation in the NEMESIS-2 sample. In the end, 3,104 individuals remained. Compared with the self-report ethnic information, 93.75% of our ethnic-QCed samples are self-reported as “Dutch”. We think some people may have understood the question as: “where were you born?”, since non-former colony countries have 0 deviations from genetic QC, while former colonies have substantial deviations. In total, 154 samples were identified as European ethnic outliers and excluded. After post-imputation quality control steps, PCA was conducted using the same SNPs (n=40,732) within NEMESIS-2 samples (n=3,104). These PCs were then used as covariates in analyses to correct for population stratification.

Proof of principle using PRSice confirmed that the correct genetic instruments were used for prediction and the genetic data significantly predicted the phenotype in the NEMESIS-2 study population (Euesden *et al.,* 2015)*.* For smoking initiation PRS, the maximum explained variance for smoking initiation was 0.79% (including non-smokers, P_T_ 5x10^-3^, p = 0.004). Regarding cigarettes per day PRS and drinks per week PRS, the explained variance of cigarettes per day was 0.54% (P_T_ 5x10^-3^, p = 1.15 x 10^-5^), and for drinks per week 1.1% (P_T_ 5x10^-7^, p = 6.47 x 10^-12^), respectively. PRS for MDD were significantly associated with a higher level of depressive symptoms using the SF-36 and MHI-3 score for all P_T_ (supplementary table S41)*.* No significant associations were found between MDD PRS and change in depressive symptoms.

**Supplementary results**

**Table S1. Number of participants changing the independent variable between baseline**

**and 3-year follow-up and 3-year and 6-year follow-up**

| **Change independent variable** | **Baseline** | **3-year follow-up** | **6-year follow-up** |
| --- | --- | --- | --- |
| No change alcohol | 0 | 3976 | 3424 |
| Quit alcohol | 0 | 23 | 18 |
| Start alcohol | 0 | 59 | 13 |
| No change smoking | 0 | 4756 | 4251 |
| Quit smoking | 0 | 307 | 231 |
| Start smoking | 0 | 153 | 136 |
| No change drinks | 0 | 2164 | 2063 |
| Increase drinks | 0 | 861 | 598 |
| Decrease drinks | 0 | 922 | 736 |
| No change cigarettes | 0 | 4231 | 3855 |
| Increase cigarettes | 0 | 384 | 317 |
| Decrease cigarettes | 0 | 594 | 443 |

**Table S2. Baseline characteristics for non-smoking vs smoking participants**

*unpaired t-test and chi-square test*

**Table S3. Baseline characteristics for non-alcohol using vs. alcohol using participants**

*unpaired t-test and chi-square test*

The mean SF-36 subscale score was 260 for non-smoking people and 255 for smoking people (p < 0.001). At baseline, the mean SF-36 subscale score was 253 for non-alcohol using and 259 for alcohol using people indicating a lower level of depressive symptoms for alcohol using people (p < 0.001).

**Mixed Linear Models corrected for age and gender**

*Multi-cross-sectional models*

Multi-cross-sectional linear mixed-model analysis showed that smoking was positively associated with depressive symptoms (estimate -0.36, standard error (se) 0.04, p-value <0.001; supplementary table 1). Similar results were found in our first sensitivity analysis using the SF-36 outcome (estimate -7.68, se 0.87, p-value <0.001, supplementary table S4).

Furthermore, we found a positive association between the mean number of cigarettes per day and depressive symptoms (6-10 cigarettes: estimate -0.22, se 0.07, p-value 0.001; 11-20 cigarettes: estimate -0.48, se 0.06, p-value <0.001; >20 cigarettes: estimate -1.00, se 0.09, p-value <0.01; table 2), also confirmed in sensitivity analysis (supplementary table S5).

Light to moderate drinking (1.5-10.5 drinks per week) was associated with less depressive symptoms (estimate 0.10, se 0.047, p 0.033; estimate 0.12, se 0.048, p 0.011), while heavier drinking (10.5-84 drinks per week) showed no significant associations (table3). Sensitivity analysis of alcohol use behaviour showed a negative association between alcohol use as a binary trait and depressive symptoms (estimate 7.77, se 2.13, p-value <0.001; supplementary table S6). Correction for additional covariates (the second sensitivity analysis) in all linear models showed that light drinking (1.5-3.5 drinks per week) was not significantly associated with depressive symptoms for the MHI-3 (supplementary table S15;S17). Furthermore, adjustment for covariates revealed no differences regarding the results for alcohol- nor smoking behaviour (see supplementary material). Sensitivity analyses thus confirmed our results.

| **Smoking** |  |  |  |
| --- | --- | --- | --- |
|  | **Participants (n = 6646)** | |  |
| Effects | Estimate | Std. Error | P |
|  | **SF-36 (3 subscales 0 - 300 points)** | |  |
| **Intercept** | **256.617** | **0.783** | **<0.001** |
| **Smoking** | **-7.677** | **0.874** | **<0.001** |
| **3-year follow-up** | **-8.793** | **0.622** | **<0.001** |
| **6-year follow-up** | **-7.973** | **0.697** | **<0.001** |
|  |  |  |  |

Table S4. Results linear mixed models multi-cross-sectional analysis: association between smoking / non - smoking and symptoms of depression using the SF-36 subscale score (corrected for age and gender).

*Fixed effects in the models were smoking, age, gender and time. Random effects were by-subject random slopes for time and intercepts for respondents. Data are estimates and P-values were calculated using the Satterthwaite approach.*

| **Cigarettes per day** |  | |  |
| --- | --- | --- | --- |
|  | **Participants (n = 6646)** | |  |
| Effects | Estimate | Std. Error | P |
|  | **SF-36 (3 subscales 0 - 300 points)** | |  |
| **Intercept** | **256.951** | **0.779** | **<0.001** |
| < 1 per week | -3.550 | 1.866 | 0.057 |
| 1-5 per day | -1.357 | 1.647 | 0.410 |
| **6-10 per day** | **-5.534** | **1.413** | **<0.001** |
| **11-20 per day** | **-9.903** | **1.248** | **<0.001** |
| **> 20 per day** | **-20.619** | **1.906** | **<0.001** |
| **3-year follow-up** | **-8.955** | **0.622** | **<0.001** |
| **6-year follow-up** | **-8.226** | **0.696** | **<0.001** |

Table S5. Results linear mixed models multi-cross-sectional analysis: association between mean number of cigarettes per day and symptoms of depression using the SF-36 subscale score (corrected for age and gender).

*Fixed effects in the models were cigarettes per day, age, gender and time. Random effects were by-subject random slopes for time and intercepts for respondents. Data are estimates and P-values were calculated using the Satterthwaite approach.*

| **Alcohol use** |  |  |  |
| --- | --- | --- | --- |
|  | **Participants (n = 6646)** |  |  |
| Effects | Estimate | Std. Error | P |
|  | **SF-36 (3 subscales 0 - 300 points)** | |  |
| **Intercept** | **246.850** | **2.151** | **<0.001** |
| **Alcohol use** | **7.770** | **2.129** | **<0.001** |
| **3-year follow-up** | **-7.232** | **0.666** | **<0.001** |
| **6-year follow-up** | **-6.622** | **0.752** | **<0.001** |
|  | **MHI-3 (3 - 18 points)** |  |  |
| **Intercept** | **15.884** | **0.103** | **<0.001** |
| Alcohol use | 0.151 | 0.101 | 0.137 |
| **3-year follow-up** | **-0.400** | **0.031** | **<0.001** |
| **6-year follow-up** | **-0.306** | **0.034** | **<0.001** |

Table S6. Results linear mixed models multi-cross-sectional analysis: association between alcohol use/ no alcohol use and symptoms of depression using the SF-36 subscale score and MHI-3 score (corrected for age and gender).

*Fixed effects in the models were alcohol use, age, gender and time. Random effects were by-subject random slopes for time and intercepts for respondents. Data are estimates and P-values were calculated using the Satterthwaite approach.*

| **Alcohol drinks per week** |  |  |  |
| --- | --- | --- | --- |
|  | **Participants (n = 6646)** | |  |
| Effects | Estimate | Std. Error | P |
|  | **SF-36 (3 subscales 0 - 300 points)** | |  |
| **Intercept** | **252.943** | **1.011** | **<0.001** |
| **drinks (1.5-3.5)** | **2.754** | **0.996** | **0.007** |
| **drinks (3.5-10.5)** | **3.614** | **1.016** | **<0.001** |
| drinks (10.5-84.0) | 0.574 | 1.104 | 0.603 |
| **3-year follow-up** | **-7.295** | **0.665** | **<0.001** |
| **6-year follow-up** | **-6.653** | **0.752** | **<0.001** |

Table S7. Results linear mixed models multi-cross-sectional analysis: association between mean number of drinks per week and symptoms of depression using the SF-36 subscale score (corrected for age and gender).

*Fixed effects in the models were number of drinks per week, age, gender and time. Random effects were by-subject random slopes for time and intercepts for respondents. Data are estimates and P-values were calculated using the Satterthwaite approach.*

| *Longitudinal models*  **Change in Smoking** | |  |  |
| --- | --- | --- | --- |
|  | **Participants (n = 6646)** | |  |
| Effects | Estimate | Std. Error | P |
|  | **SF-36 (3 subscales 0 - 300 points)** | |  |
| **Intercept** | **-7.902** | **0.741** | **<0.001** |
| Quit smoking | -0.206 | 1.818 | 0.910 |
| Start smoking | 0.102 | 2.445 | 0.967 |
| **6-year follow-up** | **9.144** | **1.083** | **<0.001** |
| **MHI-3 (3 - 18 points)** | | | |
| **Intercept** | **-0.434** | **0.034** | **<0.001** |
| Quit smoking | -0.004 | 0.083 | 0.964 |
| Start smoking | 0.051 | 0.111 | 0.649 |
| **6-year follow-up** | **0.512** | **0.049** | **<0.001** |

Table S8. Results linear mixed models longitudinal analysis: association between change in smoking and symptoms of depression using the SF-36 subscale score and MHI-3 score (corrected for age and gender).

*Fixed effects in the models were change in smoking, age, gender and time. Random effects were by-subject random slopes for time and intercepts for respondents. Data are estimates and P-values were calculated using the Satterthwaite approach.*

| **Change in cigarettes per day (binary)** | | |  |
| --- | --- | --- | --- |
|  | **Participants (n = 6646)** |  |  |
| Effects | Estimate | Std. Error | P |
|  | **SF-36 (3 subscales 0 - 300 points)** | |  |
| **Intercept** | **-8.226** | **0.758** | **<0.001** |
| Increase cigarettes | 0.302 | 1.576 | 0.848 |
| Decrease cigarettes | 1.702 | 1.319 | 0.197 |
| **6-year follow-up** | **9.232** | **1.084** | **<0.001** |
| **MHI-3 (3 - 18 points)** | | | |
| **Intercept** | **-0.439** | **0.035** | **<0.001** |
| Increase cigarettes | -0.045 | 0.072 | 0.529 |
| Decrease cigarettes | 0.063 | 0.060 | 0.293 |
| **6-year follow-up** | **0.515** | **0.049** | **<0.001** |

Table S9. Results linear mixed models longitudinal analysis: association between change in cigarettes per day (binary) and symptoms of depression using the SF-36 subscale score and MHI-3 score (corrected for age and gender).

*Fixed effects in the models were change in cigarettes per day, age, gender and time. Random effects were by-subject random slopes for time and intercepts for respondents. Data are estimates and P-values were calculated using the Satterthwaite approach.*

**Change in cigarettes per day (per level)**

|  | **Participants (n = 6646)** | |  | |
| --- | --- | --- | --- | --- |
| Effects | Estimate | Std. Error | P | |
|  | **SF-36 (3 subscales 0 - 300 points)** | |  |  |
| **Intercept** | **-8.232** | **0.758** | **<0.001** | |
| Increase 1 level | 0.448 | 1.911 | 0.815 | |
| Increase 2 levels | 2.285 | 3.857 | 0.554 | |
| Increase 3 levels | -3.316 | 5.114 | 0.517 | |
| Increase 4 levels | 0.133 | 5.683 | 0.981 | |
| Increase 5 levels | -7.668 | 13.618 | 0.573 | |
| **Decrease 1 level** | **3.357** | **1.715** | **0.050** | |
| Decrease 2 levels | -0.071 | 3.139 | 0.982 | |
| Decrease 3 levels | 0.599 | 3.878 | 0.877 | |
| Decrease 4 levels | -3.510 | 3.357 | 0.296 | |
| Decrease 5 levels | 7.538 | 6.896 | 0.274 | |
| **6-year follow-up** | **9.243** | **1.084** | **<0.001** | |
| **MHI-3 (3 - 18 points)** | | | | |
| **Intercept** | **-0.440** | **0.035** | **<0.001** | |
| Increase 1 level | -0.005 | 0.087 | 0.958 | |
| Increase 2 levels | -0.219 | 0.179 | 0.221 | |
| Increase 3 levels | 0.001 | 0.231 | 0.995 | |
| Increase 4 levels | 0.014 | 0.261 | 0.958 | |
| Increase 5 levels | -0.842 | 0.592 | 0.156 | |
| Decrease 1 level | 0.134 | 0.078 | 0.087 | |
| Decrease 2 levels | 0.024 | 0.143 | 0.869 | |
| Decrease 3 levels | 0.013 | 0.177 | 0.940 | |
| Decrease 4 levels | -0.293 | 0.154 | 0.058 | |
| **Decrease 5 levels** | **0.756** | **0.312** | **0.015** | |
| **6-year follow-up** | **0.516** | **0.049** | **<0.01** | |
|  |  |  |  | |

Table S10. Results linear mixed models longitudinal analysis: association between change in cigarettes per day (per level) and symptoms of depression using the SF-36 subscale score and MHI-3 score (corrected for age and gender).

*Fixed effects in the models were change in cigarettes per day, age, gender and time. Random effects were by-subject random slopes for time and intercepts for respondents. Data are estimates and P-values were calculated using the Satterthwaite approach.*

**Change in alcohol use**

|  | **Participants (n = 6646)** | |  |
| --- | --- | --- | --- |
| Effects | Estimate | Std. Error | P |
|  | **SF-36 (3 subscales 0 - 300 points)** | |  |
| **Intercept** | **-6.943** | **0.775** | **<0.001** |
| Quit alcohol use | 2.253 | 6.373 | 0.724 |
| Start alcohol use | -5.404 | 4.765 | 0.257 |
| **6-year follow-up** | **7.777** | **1.1787** | **<0.001** |

Table S11. Results linear mixed models longitudinal analysis: association between change in alcohol use and symptoms of depression using the SF-36 subscale score (corrected for age and gender).

*Fixed effects in the models were change in alcohol use, age, gender and time. Random effects were by-subject random slopes for time and intercepts for respondents. Data are estimates and P-values were calculated using the Satterthwaite approach.*

**Change in drinks per week (binary)**

|  | **Participants (n = 6646)** | |  |
| --- | --- | --- | --- |
| Effects | Estimate | Std. Error | P |
|  | **SF-36 (3 subscales 0 - 300 points)** | |  |
| **Intercept** | **-7.283** | **0.879** | **<0.001** |
| Increase drinks | -0.154 | 1.176 | 0.896 |
| Decrease drinks | 1.100 | 1.126 | 0.328 |
| **6-year follow-up** | **7.919** | **1.186** | **<0.001** |
| **MHI-3 (3 - 18 points)** | | | |
| **Intercept** | **-0.435** | **0.040** | **<0.001** |
| Increase drinks | -0.036 | 0.053 | 0.504 |
| Decrease drinks | 0.095 | 0.051 | 0.063 |
| **6-year follow-up** | **0.505** | **0.054** | **<0.001** |

Table S12. Results linear mixed models longitudinal analysis: association between change in drinks per week (binary) and symptoms of depression using the SF-36 subscale score and MHI-3 score (corrected for age and gender).

*Fixed effects in the models were change in drinks per week, age, gender and time. Random effects were by-subject random slopes for time and intercepts for respondents. Data are estimates and P-values were calculated using the Satterthwaite approach.*

**Change in drinks per week (per level)**

|  | **Participants (n – 6646)** | |  |
| --- | --- | --- | --- |
| Effects | Estimate | Std. Error | P |
|  | **SF-36 (3 subscales 0 - 300 points)** |  |  |
| **Intercept** | **-7.337** | **0.879** | **<0.001** |
| Increase 1 level | -0.127 | 1.260 | 0.920 |
| Increase 2 levels | -2.479 | 2.662 | 0.352 |
| **Increase 3 levels** | **13.183** | **6.568** | **0.045** |
| Decrease 1 level | 0.438 | 1.216 | 0.719 |
| Decrease 2 levels | 4.243 | 2.424 | 0.080 |
| Decrease 3 levels | 2.012 | 5.115 | 0.694 |
| **6-year follow-up** | **7.979** | **1.186** | **<0.001** |

Table S13. Results linear mixed models longitudinal analysis: association between change in drinks per week (per level) and symptoms of depression using the SF-36 subscale score (corrected for age and gender).

*Fixed effects in the models were change in drinks per week, age, gender and time. Random effects were by-subject random slopes for time and intercepts for respondents. Data are estimates and P-values were calculated using the Satterthwaite approach.*

**Mixed Linear Models corrected for additional covariates**

In our second sensitivity analysis we corrected for all covariates for both the MHI-3 and SF-36. The covariates in this second sensitivity analysis were age, gender, treatment for any psychiatric disorder in the previous 12 months, negative life events in the past or since the last interview, and cannabis dependency/abuse - all measured as binary traits; and education level, which was categorized into either primary, lower secondary, higher secondary or higher professional education. Results for this analysis are shown in supplementary table S14-S23 below. Additionally, alcohol use as a binary trait and drinks per week were entered as additional covariates to the second sensitivity analysis model for smoking status / cigarettes per day and smoking status as a binary trait and cigarettes per day for alcohol use / drinks per week to examine whether associations of smoking and alcohol use were independent of each other. The results were the same as for the analyses using the extensive set of covariates without these last two covariates (data not shown).

Multi-cross-sectional

| **Smoking** |  |  |  |
| --- | --- | --- | --- |
|  | **Participants (n = 6646)** | |  |
| Effects | Estimate | Std. Error | P |
|  | **SF-36 (3 subscales 0 - 300 points)** | |  |
| **Intercept** | **258.858** | **1.603** | **<0.001** |
| **Smoking** | **-5.334** | **0.821** | **<0.001** |
| **3-year follow-up** | **-9.532** | **0.613** | **<0.001** |
| **6-year follow-up** | **-8.404** | **0.679** | **<0.001** |
| **MHI-3 (3 - 18 points)** | | | |
| **Intercept** | **16.188** | **0.079** | **<0.001** |
| **Smoking** | **-0.272** | **0.040** | **<0.001** |
| **3-year follow-up** | **-0.461** | **0.029** | **<0.001** |
| **6-year follow-up** | **-0.353** | **0.031** | **<0.001** |
|  |  |  |  |

Table S14. Results linear mixed models multi-cross-sectional analysis: association between smoking / non - smoking and symptoms of depression using the SF-36 subscale score and MHI-3 score (corrected for age, gender, education level, negative life events, treatment for any psychiatric disorder and cannabis dependency/abuse).

*Fixed effects in the models were smoking, age, gender, education level, negative life events, treatment for any psychiatric disorder, cannabis dependency/abuse and time. Random effects were by-subject random slopes for time and intercepts for respondents. Data are estimates and P-values were calculated using the Satterthwaite approach.*

| **Cigarettes per day** |  | |  | |
| --- | --- | --- | --- | --- |
|  | **Participants (n = 6646)** | |  | |
| Effects | Estimate | Std. Error | P | |
|  | **SF-36 (3 subscales 0 - 300 points)** | |  |  |
| **Intercept** | **259.913** | **1.607** | **<0.001** | |
| **< 1 per week** | **-3.749** | **1.789** | **0.036** | |
| 1-5 per day | 1.094 | 1.569 | 0.485 | |
| **6-10 per day** | **-3.654** | **1.341** | **0.006** | |
| **11-20 per day** | **-6.772** | **1.185** | **<0.001** | |
| **> 20 per day** | **-16.281** | **1.811** | **<0.001** | |
| **3-year follow-up** | **-9.645** | **0.612** | **<0.001** | |
| **6-year follow-up** | **-8.577** | **0.678** | **<0.001** | |
|  | **MHI-3 (3 - 18 points)** |  |  | |
| **Intercept** | **16.245** | **0.080** | **<0.001** | |
| < 1 per week | -0.133 | 0.085 | 0.118 | |
| 1-5 per day | -0.038 | 0.075 | 0.611 | |
| **6-10 per day** | **-0.154** | **0.064** | **0.016** | |
| **11-20 per day** | **-0.354** | **0.057** | **<0.001** | |
| **> 20 per day** | **-0.843** | **0.087** | **<0.001** | |
| **3-year follow-up** | **-0.466** | **0.029** | **<0.001** | |
| **6-year follow-up** | **-0.361** | **0.031** | **<0.001** | |

Table S15. Results linear mixed models multi-cross-sectional analysis: association between mean number of cigarettes per day and symptoms of depression using the SF-36 subscale score and MHI-3 score (corrected for age, gender, education level, negative life events, treatment for any psychiatric disorder and cannabis dependency/abuse).

*Fixed effects in the models were cigarettes per day, age, gender, education level, negative life events, treatment for any psychiatric disorder, cannabis dependency/abuse and time. Random effects were by-subject random slopes for time and intercepts for respondents. Data are estimates and P-values were calculated using the Satterthwaite approach.*

| **Alcohol use** |  |  |  |
| --- | --- | --- | --- |
|  | **Participants (n = 6646)** |  |  |
| Effects | Estimate | Std. Error | P |
|  | **SF-36 (3 subscales 0 - 300 points)** | |  |
| **Intercept** | **253.262** | **2.457** | **<0.001** |
| **Alcohol use** | **6.311** | **2.037** | **0.002** |
| **3-year follow-up** | **-8.153** | **0.663** | **<0.001** |
| **6-year follow-up** | **-7.210** | **0.0.735** | **<0.001** |
|  | MHI-3 (3 - 18 points) |  |  |
| **Intercept** | **16.151** | **0.121** | **<0.001** |
| Alcohol use | 0.084 | 0.099 | 0.398 |
| **3-year follow-up** | **-0.430** | **0.031** | **<0.001** |
| **6-year follow-up** | **-0.323** | **0.034** | **<0.001** |

Table S16. Results linear mixed models multi-cross-sectional analysis: association between alcohol use/ no alcohol use and symptoms of depression using the SF-36 subscale score and MHI-3 score (corrected for age, gender, education level, negative life events, treatment for any psychiatric disorder and cannabis dependency/abuse).

*Fixed effects in the models were alcohol use, age, gender, education level, negative life events, treatment for any psychiatric disorder, cannabis dependency/abuse and time. Random effects were by-subject random slopes for time and intercepts for respondents. Data are estimates and P-values were calculated using the Satterthwaite approach.*

| **Alcohol drinks per week** |  |  |  |
| --- | --- | --- | --- |
|  | **Participants (n = 6646)** | |  |
| Effects | Estimate | Std. Error | P |
|  | **SF-36 (3 subscales 0 - 300 points)** | |  |
| **Intercept** | **258.241** | **1.725** | **<0.001** |
| **drinks (1.5-3.5)** | **2.389** | **0.962** | **0.013** |
| **drinks (3.5-10.5)** | **3.282** | **0.973** | **<0.001** |
| drinks (10.5-84.0) | 0.718 | 1.049 | 0.494 |
| **3-year follow-up** | **-8.231** | **0.660** | **<0.001** |
| **6-year follow-up** | **-7.265** | **0.736** | **<0.001** |
| MHI-3 (3 - 18 points) | | | |
| **Intercept** | **16.244** | **0.086** | **<0.001** |
| drinks (1.5-3.5) | 0.080 | 0.046 | 0.081 |
| **drinks (3.5-10.5)** | **0.108** | **0.047** | **0.021** |
| drinks (10.5-84.0) | -0.058 | 0.051 | 0.252 |
| **3-year follow-up** | **-0.442** | **0.031** | **<0.001** |
| **6-year follow-up** | **-0.333** | **0.034** | **<0.001** |

Table S17. Results linear mixed models multi-cross-sectional analysis: association between mean number of drinks per week and symptoms of depression using the SF-36 subscale score and MHI-3 score (corrected for age, gender, education level, negative life events, treatment for any psychiatric disorder and cannabis dependency/abuse).

*Fixed effects in the models were number of drinks per week, age, gender, education level, negative life events, treatment for any psychiatric disorder, cannabis dependency/abuse and time. Random effects were by-subject random slopes for time and intercepts for respondents. Data are estimates and P-values were calculated using the Satterthwaite approach.*

**Longitudinal models corrected for additional covariates**

| **Change in Smoking** | |  |  |
| --- | --- | --- | --- |
|  | **Participants (n = 6646)** | |  |
| Effects | Estimate | Std. Error | P |
|  | **SF-36 (3 subscales 0 - 300 points)** | |  |
| **Intercept** | **-3.459** | **1.458** | **0.018** |
| Quit smoking | -0.213 | 1.809 | 0.906 |
| Start smoking | 0.806 | 2.437 | 0.741 |
| **6-year follow-up** | **9.285** | **1.077** | **<0.001** |
| MHI-3 (3 - 18 points) | | | |
| **Intercept** | **-0.256** | **0.067** | **<0.001** |
| Quit smoking | -0.003 | 0.083 | 0.967 |
| Start smoking | 0.075 | 0.111 | 0.500 |
| **6-year follow-up** | **0.518** | **0.049** | **<0.001** |

Table S18. Results linear mixed models longitudinal analysis: association between change in smoking and symptoms of depression using the SF-36 subscale score and MHI-3 score (corrected for age, gender, education level, negative life events, treatment for any psychiatric disorder and cannabis dependency/abuse).

*Fixed effects in the models were change in smoking, age, gender, education level, negative life events, treatment for any psychiatric disorder, cannabis dependency/abuse and time. Random effects were by-subject random slopes for time and intercepts for respondents. Data are estimates and P-values were calculated using the Satterthwaite approach.*

| **Change in cigarettes per day (binary)** | | |  |
| --- | --- | --- | --- |
|  | **Participants (n = 6646)** |  |  |
| Effects | Estimate | Std. Error | P |
|  | **SF-36 (3 subscales 0 - 300 points)** | |  |
| **Intercept** | **-4.032** | **1.481** | **0.006** |
| Increase cigarettes | 0.782 | 1.575 | 0.620 |
| Decrease cigarettes | 2.145 | 1.317 | 0.103 |
| **6-year follow-up** | **9.382** | **1.078** | **<0.001** |
| **MHI-3 (3 - 18 points)** | | | |
| **Intercept** | **-0.266** | **0.068** | **<0.001** |
| Increase cigarettes | -0.031 | 0.072 | 0.663 |
| Decrease cigarettes | 0.075 | 0.060 | 0.213 |
| **6-year follow-up** | **0.521** | **0.049** | **<0.001** |

Table S19. Results linear mixed models longitudinal analysis: association between change in cigarettes per day (binary) and symptoms of depression using the SF-36 subscale score and MHI-3 score (corrected for age, gender, education level, negative life events, treatment for any psychiatric disorder and cannabis dependency/abuse).

*Fixed effects in the models were change in cigarettes per day, age, gender, education level, negative life events, treatment for any psychiatric disorder, cannabis dependency/abuse and time. Random effects were by-subject random slopes for time and intercepts for respondents. Data are estimates and P-values were calculated using the Satterthwaite approach.*

**Change in cigarettes per day (per level)**

|  | **Participants (n = 6646)** | |  | |
| --- | --- | --- | --- | --- |
| Effects | Estimate | Std. Error | P | |
|  | **SF-36 (3 subscales 0 - 300 points)** | |  |  |
| **Intercept** | **-4.036** | **1.482** | **0.006** | |
| Increase 1 level | 0.640 | 1.905 | 0.737 | |
| Increase 2 levels | 2.506 | 3.845 | 0.514 | |
| Increase 3 levels | -1.853 | 5.097 | 0.716 | |
| Increase 4 levels | 2.917 | 5.674 | 0.607 | |
| Increase 5 levels | -8.128 | 13.557 | 0.549 | |
| **Decrease 1 level** | **3.940** | **1.712** | **0.021** | |
| Decrease 2 levels | 0.127 | 3.125 | 0.968 | |
| Decrease 3 levels | 0.822 | 3.860 | 0.831 | |
| Decrease 4 levels | -3.195 | 3.343 | 0.339 | |
| Decrease 5 levels | 8.164 | 6.873 | 0.235 | |
| **6-year follow-up** | **9.391** | **1.078** | **<0.001** | |
| **MHI-3 (3 - 18 points)** | | | | |
| **Intercept** | **-0.269** | **0.068** | **<0.001** | |
| Increase 1 level | -0.001 | 0.087 | 0.991 | |
| Increase 2 levels | -0.215 | 0.178 | 0.228 | |
| Increase 3 levels | 0.049 | 0.231 | 0.834 | |
| Increase 4 levels | 0.097 | 0.261 | 0.709 | |
| Increase 5 levels | -0.864 | 0.591 | 0.144 | |
| Decrease 1 level | 0.150 | 0.078 | 0.056 | |
| Decrease 2 levels | 0.028 | 0.143 | 0.842 | |
| Decrease 3 levels | 0.023 | 0.176 | 0.898 | |
| Decrease 4 levels | -0.286 | 0.154 | 0.063 | |
| **Decrease 5 levels** | **0.773** | **0.312** | **0.013** | |
| **6-year follow-up** | **0.522** | **0.049** | **<0.001** | |
|  |  |  |  | |

Table S20. Results linear mixed models longitudinal analysis: association between change in cigarettes per day (per level) and symptoms of depression using the SF-36 subscale score and MHI-3 score (corrected for age, gender, education level, negative life events, treatment for any psychiatric disorder and cannabis dependency/abuse).

*Fixed effects in the models were change in cigarettes per day, age, gender, education level, negative life events, treatment for any psychiatric disorder, cannabis dependency/abuse and time. Random effects were by-subject random slopes for time and intercepts for respondents. Data are estimates and P-values were calculated using the Satterthwaite approach.*

**Change in alcohol use**

|  | Participants (n = 6646) | |  |
| --- | --- | --- | --- |
| Effects | Estimate | Std. Error | P |
|  | SF-36 (3 subscales 0 - 300 points) | |  |
| Intercept | -2.024 | 1.628 | 0.214 |
| Quit alcohol use | 4.146 | 6.366 | 0.515 |
| Start alcohol use | -4.393 | 4.765 | 0.357 |
| **6-year follow-up** | **7.903** | **1.175** | **<0.001** |
| **MHI-3 (3 - 18 points)** | | | |
| **Intercept** | **-0.208** | **0.074** | **0.005** |
| **Quit alcohol use** | **0.672** | **0.286** | **0.019** |
| Start alcohol use | -0.009 | 0.217 | 0.968 |
| **6-year follow-up** | **0.498** | **0.053** | **<0.001** |

Table S21. Results linear mixed models longitudinal analysis: association between change in alcohol use and symptoms of depression using the SF-36 subscale score and MHI-3 score (corrected for age, gender, education level, negative life events, treatment for any psychiatric disorder and cannabis dependency/abuse).

*Fixed effects in the models were change in alcohol use, age, gender, education level, negative life events, treatment for any psychiatric disorder, cannabis dependency/abuse and time. Random effects were by-subject random slopes for time and intercepts for respondents. Data are estimates and P-values were calculated using the Satterthwaite approach.*

**Change in drinks per week (binary)**

|  | **Participants (n = 6646)** | |  |
| --- | --- | --- | --- |
| Effects | Estimate | Std. Error | P |
|  | **SF-36 (3 subscales 0 - 300 points)** | |  |
| Intercept | -2.853 | 1.708 | 0.095 |
| Increase drinks | -0.057 | 1.174 | 0.961 |
| Decrease drinks | 1.097 | 1.124 | 0.329 |
| **6-year follow-up** | **8.027** | **1.181** | **<0.001** |
| **MHI-3 (3 - 18 points)** | | | |
| **Intercept** | **-0.229** | **0.078** | **0.003** |
| Increase drinks | -0.034 | 0.053 | 0.523 |
| Decrease drinks | 0.093 | 0.051 | 0.070 |
| **6-year follow-up** | **0.509** | **0.054** | **<0.001** |

Table S22. Results linear mixed models longitudinal analysis: association between change in drinks per week (binary) and symptoms of depression using the SF-36 subscale score and MHI-3 score (corrected for age, gender, education level, negative life events, treatment for any psychiatric disorder and cannabis dependency/abuse).

*Fixed effects in the models were change in drinks per week, age, gender, education level, negative life events, treatment for any psychiatric disorder, cannabis dependency/abuse and time. Random effects were by-subject random slopes for time and intercepts for respondents. Data are estimates and P-values were calculated using the Satterthwaite approach.*

**Change in drinks per week (per level)**

|  | **Participants (n – 6646)** | |  |
| --- | --- | --- | --- |
| Effects | Estimate | Std. Error | P |
|  | **SF-36 (3 subscales 0 - 300 points)** |  |  |
| Intercept | -2.956 | 1.710 | 0.084 |
| Increase 1 level | -0055 | 1.257 | 0.965 |
| Increase 2 levels | -2.240 | 2.664 | 0.400 |
| **Increase 3 levels** | **13.243** | **6.571** | **0.044** |
| Decrease 1 level | 0.400 | 1.214 | 0.742 |
| Decrease 2 levels | 4.083 | 2.421 | 0.092 |
| Decrease 3 levels | 3.608 | 5.115 | 0.481 |
| **6-year follow-up** | **8.089** | **1.182** | **<0.001** |
| **MHI-3 (3 - 18 points)** | | | |
| **Intercept** | **-0.233** | **0.078** | **0.003** |
| Increase 1 level | -0.011 | 0.057 | 0.843 |
| Increase 2 levels | -0.201 | 0.121 | 0.096 |
| Increase 3 levels | 0.149 | 0.301 | 0.620 |
| Decrease 1 level | 0.029 | 0.055 | 0.605 |
| **Decrease 2 levels** | **0.347** | **0.110** | **0.002** |
| **Decrease 3 levels** | **0.461** | **0.233** | **0.048** |
| **6-year follow-up** | **0.512** | **0.054** | **<0.001** |

Table S23. Results linear mixed models longitudinal analysis: association between change in drinks per week (per level) and symptoms of depression using the SF-36 subscale score and MHI-3 score (corrected for age, gender, education level, negative life events, treatment for any psychiatric disorder and cannabis dependency/abuse)

*Fixed effects in the models were change in drinks per week, age, gender, education level, negative life events, treatment for any psychiatric disorder, cannabis dependency/abuse and time. Random effects were by-subject random slopes for time and intercepts for respondents. Data are estimates and P-values were calculated using the Satterthwaite approach.*

**Mixed Linear Models corrected for age and gender – validation drinks per week**

At last, we validated our findings for drinks per week and repeated the multi-cross-sectional and longitudinal analyses using different thresholds for males and females. Drinks per week were recategorized for women (i.e. 0-1, 1-14, 14-84 drinks per week) and men (i.e. 0-1, 1-21, 21-84 drinks per week). Results are shown in table S24-S37 below.

*Descriptive statistics*

**Table S24. Baseline independent variable drinks per week**

| **Drinks per week** | **Baseline** | | **3-year follow-up** | **6-year follow-up** |  |
| --- | --- | --- | --- | --- | --- |
| **Females** |  | |  |  |  |
| *[0-1]* | 1093 | | 579 | 474 |  |
| *[1-14]* | 1681 | | 1398 | 1261 |  |
| *[14-84]* | 170 | | 122 | 102 |  |
| **Males** |  | |  |  |  |
| *[0-1]* | 551 | | 287 | 265 |  |
| *[1-21]* | 1946 | | 1632 | 1400 |  |
| *[21-84]* | 209 | | 137 | 114 |  |
|  | |  |  |  |  |

**Table S25. Number of participants changing the independent variable drinks per week between baseline and 3-year follow-up and 3-year and 6-year follow-up**

| **Change in drinks per week** | **Baseline** | **3-year follow-up** | **6-year follow-up** |
| --- | --- | --- | --- |
| **Females** |  |  |  |
| No change | 0 | 1444 | 1338 |
| Increase 1 level | 0 | 269 | 181 |
| Increase 2 levels | 0 | 4 | 0 |
| Decrease 1 level | 0 | 252 | 192 |
| Decrease 2 levels | 0 | 5 | 0 |
| **Males** |  |  |  |
| No change | 0 | 1578 | 1393 |
| Increase 1 level | 0 | 193 | 122 |
| Increase 2 levels | 0 | 2 | 1 |
| Decrease 1 level | 0 | 195 | 167 |
| Decrease 2 levels | 0 | 4 | 3 |

**Females**

*Multi cross sectional*

| **Alcohol drinks per week** |  |  |  | |
| --- | --- | --- | --- | --- |
|  | **Participants (n = 6646)** | |  | |
| Effects | Estimate | Std. Error | P | |
| **Females** |  |  |  | |
|  | **SF-36 (3 subscales 0 - 300 points)** | |  | |
| **Intercept** | **246.591** | **1.096** | **<0.001** | |
| drinks (1-14) | 0.812 | 1.170 | 0.488 | |
| **drinks (14-84)** | **-7.460** | **2.466** | **0.002** | |
| **3-year follow-up** | **-7.737** | **0.993** | **<0.001** | |
| **6-year follow-up** | **-7.248** | **1.115** | **<0.001** | |
|  | **MHI-3 (3 - 18 points)** | | | |
| **Intercept** | **15.847** | **0.050** | **<0.001** |  |
| drinks (1-14) | 0.036 | 0.053 | 0.484 |  |
| **drinks (14-84.0)** | **-0.286** | **0.111** | **0.010** |  |
| **3-year follow-up** | **-0.396** | **0.043** | **<0.001** |  |
| **6-year follow-up** | **-0.316** | **0.050** | **<0.001** |  |

Table S26. Results linear mixed models multi-cross-sectional analysis: association between mean number of drinks per week and symptoms of depression using the SF-36 subscale score and MHI-3 score (corrected for age and gender) in females.

*Fixed effects in the models were number of drinks per week, age, gender and time. Random effects were by-subject random slopes for time and intercepts for respondents. Data are estimates and P-values were calculated using the Satterthwaite approach.*

*Longitudinal*

**Change in drinks per week (binary)**

|  | **Participants (n = 6646)** | | |  |
| --- | --- | --- | --- | --- |
| Effects | Estimate | | Std. Error | P |
|  | **SF-36 (3 subscales 0 - 300 points)** | | |  |
| **Intercept** | **-7.993** | | **1.108** | **<0.001** |
| Increase drinks | -1.279 | | 2.119 | 0.546 |
| **Decrease drinks** | **4.851** | | **2.114** | **0.022** |
| **6-year follow-up** | **8.365** | | **1.765** | **<0.001** |
|  | | **MHI-3 (3 - 18 points)** | | |
| **Intercept** | **-0.449** | | **0.048** | **<0.001** |
| Increase drinks | 0.097 | | 0.093 | 0.298 |
| **Decrease drinks** | **0.225** | | **0.093** | **0.016** |
| **6-year follow-up** | **0.472** | | **0.076** | **<0.001** |

Table S27. Results linear mixed models longitudinal analysis: association between change in drinks per week (binary) and symptoms of depression using the SF-36 subscale score and MHI-3 score (corrected for age and gender) in females.

*Fixed effects in the models were change in drinks per week, age, gender and time. Random effects were by-subject random slopes for time and intercepts for respondents. Data are estimates and P-values were calculated using the Satterthwaite approach.*

**Change in drinks per week (per level)**

|  | **Participants (n – 6646)** | | |  |
| --- | --- | --- | --- | --- |
| Effects | Estimate | Std. Error | | P |
|  | **SF-36 (3 subscales 0 - 300 points)** |  | |  |
| **Intercept** | **-7.993** | **1.109** | | **<0.001** |
| Increase 1 level | -1.437 | 2.127 | | 0.499 |
| Increase 2 levels | 17.624 | 20.463 | | 0.389 |
| **Decrease 1 level** | **4.877** | **2.124** | | **0.022** |
| Decrease 2 levels | -3.273 | 19.116 | | 0.864 |
| **6-year follow-up** | **8.377** | **1.767** | | **<0.001** |
|  | **MHI-3 (3 - 18 points)** | | | |
| **Intercept** | **-0.450** | **0.048** |  | **<0.001** |
| Increase 1 level | 0.093 | 0.094 | | 0.324 |
| Increase 2 levels | 0.474 | 0.911 | | 0.603 |
| **Decrease 1 level** | **0.211** | **0.094** | | **0.025** |
| Decrease 2 levels | 1.441 | 0.845 | | 0.088 |
| **6-year follow-up** | **0.475** | **0.076** | | **<0.001** |

Table S28. Results linear mixed models longitudinal analysis: association between change in drinks per week (per level) and symptoms of depression using the SF-36 subscale score (corrected for age and gender) in females.

*Fixed effects in the models were change in drinks per week, age, gender and time. Random effects were by-subject random slopes for time and intercepts for respondents. Data are estimates and P-values were calculated using the Satterthwaite approach.*

**Males**

*Multi cross sectional*

| **Alcohol drinks per week** |  |  |  |
| --- | --- | --- | --- |
|  | **Participants (n = 6646)** | |  |
| Effects | Estimate | Std. Error | P |
|  | **SF-36 (3 subscales 0 - 300 points)** | |  |
| **Intercept** | **250.527** | **1.276** | **<0.001** |
| **drinks (1-21)** | **5.857** | **1.293** | **<0.001** |
| **drinks (21-84.0)** | **-5.271** | **2.170** | **0.015** |
| **3-year follow-up** | **-6.978** | **0.879** | **<0.001** |
| **6-year follow-up** | **-6.137** | **0.996** | **<0.001** |
|  | **MHI-3 (3 - 18 points)** | | |
| **Intercept** | **15.948** | **0.064** | **<0.001** |
| **drinks (1-14)** | **0.164** | **0.064** | **0.010** |
| **drinks (14-84.0)** | **-0.277** | **0.107** | **0.010** |
| **3-year follow-up** | **-0.425** | **0.044** | **<0.001** |
| **6-year follow-up** | **-0.310** | **0.047** | **<0.001** |
|  |  |  |  |

Table S29. Results linear mixed models multi-cross-sectional analysis: association between mean number of drinks per week and symptoms of depression using the SF-36 subscale score and MHI-3 score (corrected for age and gender).

*Fixed effects in the models were number of drinks per week, age, gender and time. Random effects were by-subject random slopes for time and intercepts for respondents. Data are estimates and P-values were calculated using the Satterthwaite approach.*

*Longitudinal*

**Change in drinks per week (binary)**

|  | **Participants (n = 6646)** | |  |
| --- | --- | --- | --- |
| Effects | Estimate | Std. Error | P |
|  | **SF-36 (3 subscales 0 - 300 points)** | |  |
| Intercept | -6.892 | 0.944 | <0.001 |
| Increase drinks | -1.311 | 2.153 | 0.543 |
| Decrease drinks | 1.613 | 2.009 | 0.422 |
| 6-year follow-up | 7.521 | 1.582 | <0.001 |
| **MHI-3 (3 - 18 points)** | | | |
| **Intercept** | **-0.460** | **0.047** | **<0.001** |
| Increase drinks | -0.020 | 0.101 | 0.843 |
| **Decrease drinks** | **0.237** | **0.094** | **0.012** |
| **6-year follow-up** | **0.541** | **0.076** | **<0.001** |

Table S30. Results linear mixed models longitudinal analysis: association between change in drinks per week (binary) and symptoms of depression using the SF-36 subscale score and MHI-3 score (corrected for age and gender).

*Fixed effects in the models were change in drinks per week, age, gender and time. Random effects were by-subject random slopes for time and intercepts for respondents. Data are estimates and P-values were calculated using the Satterthwaite approach.*

**Change in drinks per week (per level)**

|  | **Participants (n – 6646)** | | |  |
| --- | --- | --- | --- | --- |
| Effects | Estimate | Std. Error | | P |
|  | **SF-36 (3 subscales 0 - 300 points)** |  | |  |
| **Intercept** | **-6.883** | **0.943** | | **<0.001** |
| Increase 1 level | -1.801 | 2.160 | | 0.404 |
| Increase 2 levels | 36.516 | 20.336 | | 0.073 |
| Decrease 1 level | 0.951 | 2.021 | | 0.638 |
| **Decrease 2 levels** | **43.898** | **14.988** | | **0.003** |
| **6-year follow-up** | **7.527** | **1.580** | | **<0.001** |
|  | **MHI-3 (3 - 18 points)** | | | |
| **Intercept** | **-0.460** | **0.046** |  | **<0.001** |
| Increase 1 level | -0.027 | 0.101 | | 0.793 |
| Increase 2 levels | -0.296 | 0.957 | | 0.757 |
| **Decrease 1 level** | **0.197** | **0.095** | | **0.038** |
| **Decrease 2 levels** | **2.438** | **0.646** | | **<0.001** |
| **6-year follow-up** | **0.542** | **0.076** | | **<0.001** |

Table S31. Results linear mixed models longitudinal analysis: association between change in drinks per week (per level) and symptoms of depression using the SF-36 subscale score (corrected for age and gender).

*Fixed effects in the models were change in drinks per week, age, gender and time. Random effects were by-subject random slopes for time and intercepts for respondents. Data are estimates and P-values were calculated using the Satterthwaite approach.*

**Mixed Linear Models corrected for additional covariates – validation drinks per week**

**Females**

*Multi cross sectional*

| **Alcohol drinks per week** |  |  |  |
| --- | --- | --- | --- |
|  | **Participants (n = 6646)** | |  |
| Effects | Estimate | Std. Error | P |
|  | **SF-36 (3 subscales 0 - 300 points)** | |  |
| **Intercept** | **252.855** | **2.358** | **<0.001** |
| drinks (1-14) | 0.807 | 1.122 | 0.472 |
| **drinks (14-84.0)** | **-4.966** | **2.362** | **0.036** |
| **3-year follow-up** | **-9.027** | **0.985** | **<0.001** |
| **6-year follow-up** | **-8.143** | **1.099** | **<0.001** |
|  | **MHI-3 (3 - 18 points)** | | |
| **Intercept** | **16.167** | **0.112** | **<0.001** |
| drinks (1-14) | 0.030 | 0.051 | 0.566 |
| **drinks (14-84.0)** | **-0.221** | **0.108** | **0.042** |
| **3-year follow-up** | **-0.442** | **0.044** | **<0.001** |
| **6-year follow-up** | **-0.344** | **0.050** | **<0.001** |
|  |  |  |  |

Table S32. Results linear mixed models multi-cross-sectional analysis: association between mean number of drinks per week and symptoms of depression using the SF-36 subscale score and MHI-3 score (corrected for age, gender, education level, negative life events, treatment for any psychiatric disorder and cannabis dependency/abuse) in females.

*Fixed effects in the models were number of drinks per week, age, gender, education level, negative life events, treatment for any psychiatric disorder, cannabis dependency/abuse and time. Random effects were by-subject random slopes for time and intercepts for respondents. Data are estimates and P-values were calculated using the Satterthwaite approach.*

*Longitudinal*

**Change in drinks per week (binary)**

|  | **Participants (n = 6646)** | |  |
| --- | --- | --- | --- |
| Effects | Estimate | Std. Error | P |
|  | **SF-36 (3 subscales 0 - 300 points)** | |  |
| Intercept | -2.969 | 2.439 | 0.223 |
| Increase drinks | -1.154 | 2.117 | 0.586 |
| **Decrease drinks** | **5.175** | **2.114** | **0.014** |
| **6-year follow-up** | **8.494** | **1.759** | **<0.001** |
| **MHI-3 (3 - 18 points)** | | | |
| **Intercept** | **-0.231** | **0.109** | **0.033** |
| Increase drinks | 0.101 | 0.093 | 0.276 |
| **Decrease drinks** | **0.234** | **0.093** | **0.012** |
| **6-year follow-up** | **0.478** | **0.076** | **<0.001** |

Table S33. Results linear mixed models longitudinal analysis: association between change in drinks per week (binary) and symptoms of depression using the SF-36 subscale score and MHI-3 score (corrected for age, gender, education level, negative life events, treatment for any psychiatric disorder and cannabis dependency/abuse) in females.

*Fixed effects in the models were change in drinks per week, age, gender, education level, negative life events, treatment for any psychiatric disorder, cannabis dependency/abuse and time. Random effects were by-subject random slopes for time and intercepts for respondents. Data are estimates and P-values were calculated using the Satterthwaite approach.*

**Change in drinks per week (per level)**

|  | **Participants (n – 6646)** | | |  |
| --- | --- | --- | --- | --- |
| Effects | Estimate | Std. Error | | P |
|  | **SF-36 (3 subscales 0 - 300 points)** |  | |  |
| Intercept | -2.959 | 2.439 | | 0.225 |
| Increase 1 level | -1.301 | 2.125 | | 0.541 |
| Increase 2 levels | 16.016 | 20.437 | | 0.433 |
| **Decrease 1 level** | **5.194** | **2.123** | | **0.015** |
| Decrease 2 levels | -1.980 | 19.066 | | 0.917 |
| **6-year follow-up** | **8.506** | **1.761** | | **<0.001** |
|  | **MHI-3 (3 - 18 points)** | | | |
| **Intercept** | **-0.232** | **0.109** |  | **0.033** |
| Increase 1 level | 0.098 | 0.094 | | 0.298 |
| Increase 2 levels | 0.394 | 0.910 | | 0.665 |
| **Decrease 1 level** | **0.220** | **0.094** | | **0.019** |
| Decrease 2 levels | 1.484 | 0.842 | | 0.078 |
| **6-year follow-up** | **0.482** | **0.076** | | **<0.001** |

Table S34. Results linear mixed models longitudinal analysis: association between change in drinks per week (per level) and symptoms of depression using the SF-36 subscale score and MHI-3 score (corrected for age, gender, education level, negative life events, treatment for any psychiatric disorder and cannabis dependency/abuse) in females.

*Fixed effects in the models were change in drinks per week, age, gender, education level, negative life events, treatment for any psychiatric disorder, cannabis dependency/abuse and time. Random effects were by-subject random slopes for time and intercepts for respondents. Data are estimates and P-values were calculated using the Satterthwaite approach.*

**Males**

*Multi cross sectional*

| **Alcohol drinks per week** |  |  |  |
| --- | --- | --- | --- |
|  | **Participants (n = 6646)** | |  |
| Effects | Estimate | Std. Error | P |
|  | **SF-36 (3 subscales 0 - 300 points)** | |  |
| **Intercept** | **257.067** | **2.276** | **<0.001** |
| **drinks (1-21)** | **4.987** | **1.239** | **<0.001** |
| **drinks (21-84.0)** | **-5.253** | **2.074** | **0.011** |
| **3-year follow-up** | **-7.531** | **0.874** | **<0.001** |
| **6-year follow-up** | **-6.457** | **0.966** | **<0.001** |
|  | **MHI-3 (3 - 18 points)** | | |
| **Intercept** | **16.189** | **0.120** | **<0.001** |
| **drinks (1-14)** | **0.134** | **0.062** | **0.032** |
| **drinks (14-84.0)** | **-0.282** | **0.105** | **0.007** |
| **3-year follow-up** | **-0.439** | **0.044** | **<0.001** |
| **6-year follow-up** | **-0.317** | **0.046** | **<0.001** |
|  |  |  |  |

Table S35. Results linear mixed models multi-cross-sectional analysis: association between mean number of drinks per week and symptoms of depression using the SF-36 subscale score and MHI-3 score (corrected for age, gender, education level, negative life events, treatment for any psychiatric disorder and cannabis dependency/abuse).

*Fixed effects in the models were number of drinks per week, age, gender, education level, negative life events, treatment for any psychiatric disorder, cannabis dependency/abuse and time. Random effects were by-subject random slopes for time and intercepts for respondents. Data are estimates and P-values were calculated using the Satterthwaite approach.*

*Longitudinal*

**Change in drinks per week (binary)**

|  | **Participants (n = 6646)** | |  |
| --- | --- | --- | --- |
| Effects | Estimate | Std. Error | P |
|  | **SF-36 (3 subscales 0 - 300 points)** | |  |
| Intercept | -3.063 | 2.177 | 0.160 |
| Increase drinks | -0.876 | 2.159 | 0.685 |
| Decrease drinks | 2.112 | 2.010 | 0.293 |
| **6-year follow-up** | **7.625** | **1.574** | **<0.001** |
| **MHI-3 (3 - 18 points)** | | | |
| **Intercept** | **-0.253** | **0.101** | **0.013** |
| Increase drinks | -0.027 | 0.102 | 0.793 |
| **Decrease drinks** | **0.234** | **0.094** | **0.013** |
| **6-year follow-up** | **0.544** | **0.076** | **<0.001** |

Table S36. Results linear mixed models longitudinal analysis: association between change in drinks per week (binary) and symptoms of depression using the SF-36 subscale score and MHI-3 score (corrected for age, gender, education level, negative life events, treatment for any psychiatric disorder and cannabis dependency/abuse).

*Fixed effects in the models were change in drinks per week, age, gender, education level, negative life events, treatment for any psychiatric disorder, cannabis dependency/abuse and time. Random effects were by-subject random slopes for time and intercepts for respondents. Data are estimates and P-values were calculated using the Satterthwaite approach.*

**Change in drinks per week (per level)**

|  | **Participants (n – 6646)** | | | |  |
| --- | --- | --- | --- | --- | --- |
| Effects | Estimate | | Std. Error | | P |
|  | **SF-36 (3 subscales 0 - 300 points)** | |  | |  |
| Intercept | -3.114 | | 2.174 | | 0.152 |
| Increase 1 level | -1.367 | | 2.164 | | 0.527 |
| **Increase 2 levels** | **42.842** | | **20.530** | | **0.037** |
| Decrease 1 level | 1.437 | | 2.019 | | 0.477 |
| **Decrease 2 levels** | **47.980** | | **14.965** | | **0.001** |
| **6-year follow-up** | **7.628** | | **1.572** | | **<0.001** |
|  | **MHI-3 (3 - 18 points)** | | | | |
| **Intercept** | **-0.255** |  | **0.102** |  | **0.012** |
| Increase 1 level | -0.032 | | 0.102 | | 0.757 |
| Increase 2 levels | -0.278 | | 0.967 | | 0.774 |
| **Decrease 1 level** | **0.195** | | **0.095** | | **0.040** |
| **Decrease 2 levels** | **2.488** | | **0.650** | | **<0.001** |
| **6-year follow-up** | **0.546** | | **0.076** | | **<0.001** |

Table S37. Results linear mixed models longitudinal analysis: association between change in drinks per week (per level) and symptoms of depression using the SF-36 subscale score and MHI-3 score (corrected for age, gender, education level, negative life events, treatment for any psychiatric disorder and cannabis dependency/abuse).

*Fixed effects in the models were change in drinks per week, age, gender, education level, negative life events, treatment for any psychiatric disorder, cannabis dependency/abuse and time. Random effects were by-subject random slopes for time and intercepts for respondents. Data are estimates and P-values were calculated using the Satterthwaite approach.*

**PRS models**

PRS CPD prediction on SF-36 and MHI-3

(*smokers and non-smokers)*

| **SF-36** | | | |
| --- | --- | --- | --- |
| pt | Estimate | SE | P-values  Satterthwaite |
| 5em8 | 0.590 | 3.591 | 0.870 |
| 5em7 | 0.108 | 3.382 | 0.975 |
| 5em6 | -0.903 | 3.013 | 0.764 |
| 5em5 | -0.683 | 2.445 | 0.780 |
| 5em4 | -2.379 | 1.734 | 0.170 |
| 5em3 | -2.480 | 0.943 | 0.009 |
| 0p05 | -1.112 | 0.465 | 0.017 |
| 0p1 | -0.885 | 0.388 | 0.022 |
| 0p2 | -0.640 | 0.329 | 0.052 |
| 0p3 | -0.655 | 0.309 | 0.034 |
| 0p4 | -0.618 | 0.298 | 0.038 |
| 0p5 | -0.633 | 0.293 | 0.031 |
| **MHI-3** | | | |
| pt | Estimate | SE | P-values  Satterthwaite |
| 5em8 | 0.205 | 0.169 | 0.225 |
| 5em7 | 0.147 | 0.159 | 0.357 |
| 5em6 | 0.133 | 0.142 | 0.349 |
| 5em5 | 0.128 | 0 .115 | 0.266 |
| 5em4 | 0.017 | 0.082 | 0.837 |
| 5em3 | -0.026 | 0.044 | 0.553 |
| 0p05 | -0.026 | 0.022 | 0.231 |
| 0p1 | -0.017 | 0.018 | 0.341 |
| 0p2 | -0.007 | 0.016 | 0.632 |
| 0p3 | -0.010 | 0.015 | 0.506 |
| 0p4 | -0.009 | 0.014 | 0.530 |
| 0p5 | -0.011 | 0.014 | 0.431 |

Table S38. Results linear mixed models PRS analysis: association between polygenic risk scores for cigarettes per day and symptoms of depression using the SF-36 subscale score and MHI-3 score (corrected for age and gender) in smokers and non-smokers.

*Fixed effects in the models were polygenic risk score for cigarettes per day, PC1, PC2, PC3, age, gender and time. Random effects were by-subject random slopes for time and intercepts for respondents. Data are estimates and P-values were calculated using the Satterthwaite approach and adjusted for multiple testing using the Benjamini & Hochberg method.*

PRS DPW prediction on SF-36 and MHI-3

| **SF-36** | | | |
| --- | --- | --- | --- |
| pt | Estimate | SE | P-values  Satterthwaite |
| 5em8 | 7.024 | 5.938 | 0.237 |
| 5em7 | 2.849 | 4.926 | 0.563 |
| 5em6 | 0.043 | 4.105 | 0.992 |
| 5em5 | 1.120 | 3.202 | 0.727 |
| 5em4 | -0.137 | 2.168 | 0.950 |
| 5em3 | 0.098 | 1.246 | 0.937 |
| 0p05 | 0.077 | 0.634 | 0.904 |
| 0p1 | 0.264 | 0.529 | 0.618 |
| 0p2 | 0.171 | 0.456 | 0.708 |
| 0p3 | 0.096 | 0.429 | 0.823 |
| 0p4 | 0.129 | 0.416 | 0.757 |
| 0p5 | 0.108 | 0.410 | 0.793 |
| **MHI-3** | | | |
| pt | Estimate | SE | P-values  Satterthwaite |
| 5em8 | -0.014 | 0.280 | 0.960 |
| 5em7 | -0.186 | 0.232 | 0.194 |
| 5em6 | -0.192 | 0.193 | 0.321 |
| 5em5 | -0.139 | 0.151 | 0.356 |
| 5em4 | -0.113 | 0.102 | 0.268 |
| 5em3 | -0.027 | 0.059 | 0.649 |
| 0p05 | -0.017 | 0.030 | 0.560 |
| 0p1 | -0.003 | 0.025 | 0.912 |
| 0p2 | 0.002 | 0.021 | 0.920 |
| 0p3 | -0.003 | 0.020 | 0.869 |
| 0p4 | -0.002 | 0.020 | 0.927 |
| 0p5 | -0.002 | 0.019 | 0.902 |

Table S39. Results linear mixed models PRS analysis: association between polygenic risk scores for drinks per week and symptoms of depression using the SF-36 subscale score and MHI-3 score (corrected for age and gender).

*Fixed effects in the models were polygenic risk score for drinks per week, PC1, PC2, PC3, age, gender and time. Random effects were by-subject random slopes for time and intercepts for respondents. Data are estimates and P-values were calculated using the Satterthwaite approach and adjusted for multiple testing using the Benjamini & Hochberg method.*

PRS SI prediction on SF-36 and MHI-3

| **SF-36** | | | |
| --- | --- | --- | --- |
| pt | Estimate | SE | P-values  Satterthwaite |
| 5em8 | -5.546 | 7.199 | 0.441 |
| 5em7 | -1.422 | 5.738 | 0.804 |
| 5em6 | 3.043 | 4.111 | 0.459 |
| 5em5 | 3.730 | 3.027 | 0.218 |
| 5em4 | 2.621 | 1.983 | 0.186 |
| 5em3 | -1.007 | 1.104 | 0.362 |
| 0p05 | -0.938 | 0.608 | 0.123 |
| 0p1 | -0.537 | 0.523 | 0.304 |
| 0p2 | -0.612 | 0.459 | 0.182 |
| 0p3 | -0.524 | 0.434 | 0.227 |
| 0p4 | -0.506 | 0.422 | 0.231 |
| 0p5 | -0.534 | 0.416 | 0.200 |
| **MHI-3** | | | |
| pt | Estimate | SE | P-values  Satterthwaite |
| 5em8 | -0.128 | 0.339 | 0.706 |
| 5em7 | 0.156 | 0.270 | 0.565 |
| 5em6 | 0.350 | 0.194 | 0.070 |
| 5em5 | 0.281 | 0.143 | 0.049 |
| 5em4 | 0.183 | 0.093 | 0.049 |
| 5em3 | -0.051 | 0.052 | 0.329 |
| 0p05 | -0.030 | 0.029 | 0.302 |
| 0p1 | -0.006 | 0.025 | 0.817 |
| 0p2 | -0.016 | 0.022 | 0.456 |
| 0p3 | -0.013 | 0.020 | 0.524 |
| 0p4 | -0.015 | 0.020 | 0.445 |
| 0p5 | -0.016 | 0.020 | 0.428 |

Table S40. Results linear mixed models PRS analysis: association between polygenic risk scores for smoking initiation and symptoms of depression using the SF-36 subscale score and MHI-3 score (corrected for age and gender).

*Fixed effects in the models were polygenic risk score for smoking initiation, PC1, PC2, PC3, age, gender and time. Random effects were by-subject random slopes for time and intercepts for respondents. Data are estimates and P-values were calculated using the Satterthwaite approach and adjusted for multiple testing using the Benjamini & Hochberg method.*

PRS MDD on SF-36 and MHI-3

| **SF-36** | | | |
| --- | --- | --- | --- |
| pt | Estimate | SE | P-values  Satterthwaite |
| 5em8 | -9.422 | 4.357 | 0.031 |
| 5em7 | -5.937 | 3.263 | 0.069 |
| 5em6 | -5.594 | 2.535 | 2.738 |
| 5em5 | -5.511 | 1.857 | 0.003 |
| 5em4 | -4.918 | 1.249 | 8.442653e-05 |
| 5em3 | -3.721 | 0.782 | 2.055612e-06 |
| 0p05 | -2.912 | 0.625 | 3.304509e-06 |
| 0p1 | -2.912 | 0.625 | 3.304509e-06 |
| 0p2 | -2.912 | 0.625 | 3.304509e-06 |
| 0p3 | -2.912 | 0.625 | 3.304509e-06 |
| 0p4 | -2.912 | 0.625 | 3.304509e-06 |
| 0p5 | -2.912 | 0.625 | 3.304509e-06 |
| MHI-3 | | | |
| pt | Estimate | SE | P-values  Satterthwaite |
| 5em8 | -0.522 | 0.205 | 0.011 |
| 5em7 | -0.353 | 0.154 | 0.022 |
| 5em6 | -0.325 | 0.119 | 0.007 |
| 5em5 | -0.255 | 0.087 | 0.004 |
| 5em4 | -0.232 | 0.059 | 8.034458e-05 |
| 5em3 | -0.156 | 0.037 | 2.399406e-05 |
| 0p05 | -0.111 | 0.029 | 1.688747e-04 |
| 0p1 | -0.111 | 0.029 | 1.688747e-04 |
| 0p2 | -0.111 | 0.029 | 1.688747e-04 |
| 0p3 | -0.111 | 0.029 | 1.688747e-04 |
| 0p4 | -0.111 | 0.029 | 1.688747e-04 |
| 0p5 | -0.111 | 0.029 | 1.688747e-04 |

Table S41. Results linear mixed models PRS analysis: association between polygenic risk scores for major depressive disorder and symptoms of depression using the SF-36 subscale score and MHI-3 score (corrected for age and gender).

*Fixed effects in the models were polygenic risk score for major depressive disorder, PC1, PC2, PC3, age, gender and time. Random effects were by-subject random slopes for time and intercepts for respondents. Data are estimates and P-values were calculated using the Satterthwaite approach and adjusted for multiple testing using the Benjamini & Hochberg method.*

PRS CPD on change in SF-36 and change in MHI-3 (*smokers and non-smokers)*

| **Change in SF-36** | | | |
| --- | --- | --- | --- |
| pt | Estimate | SE | P-values  Satterthwaite |
| 5em8 | 2.313 | 2.692 | 0.390 |
| 5em7 | 2.402 | 2.539 | 0.344 |
| 5em6 | 2.204 | 2.259 | 0.329 |
| 5em5 | 0.126 | 1.842 | 0.946 |
| 5em4 | 0.329 | 1.303 | 0.801 |
| 5em3 | 0.108 | 0.711 | 0.879 |
| 0p05 | 0.0245 | 0.351 | 0.944 |
| 0p1 | -0.180 | 0.290 | 0.535 |
| 0p2 | -0.184 | 0.247 | 0.457 |
| 0p3 | -0.066 | 0.232 | 0.776 |
| 0p4 | -0.027 | 0.224 | 0.904 |
| 0p5 | -0.039 | 0.220 | 0.861 |
| **Change in MHI-3** | | | |
| pt | Estimate | SE | P-values  Satterthwaite |
| 5em8 | -0.452 | 0.119 | 0.308 |
| 5em7 | 0.125 | 0.112 | 0.265 |
| 5em6 | 0.101 | 0.100 | 0.313 |
| 5em5 | 0.023 | 0.082 | 0.778 |
| 5em4 | -0.049 | 0.058 | 0.392 |
| 5em3 | -0.061 | 0.031 | 0.052 |
| 0p05 | -0.015 | 0.016 | 0.343 |
| 0p1 | -0.012 | 0.013 | 0.345 |
| 0p2 | -0.010 | 0.011 | 0.375 |
| 0p3 | -0.008 | 0.010 | 0.424 |
| 0p4 | -0.007 | 0.010 | 0.490 |
| 0p5 | -0.007 | 0.010 | 0.483 |

Table S42. Results linear mixed models PRS analysis: association between polygenic risk scores for cigarettes per day and change in symptoms of depression using the SF-36 subscale score and MHI-3 score (corrected for age and gender) in smokers and non-smokers.

*Fixed effects in the models were polygenic risk score for cigarettes per day, PC1, PC2, PC3, age, gender and time. Random effects were by-subject random slopes for time and intercepts for respondents. Data are estimates and P-values were calculated using the Satterthwaite approach and adjusted for multiple testing using the Benjamini & Hochberg method.*

PRS CPD on change in SF-36 and change in MHI-3 (*smokers)*

| **Change in SF-36** | | | |
| --- | --- | --- | --- |
| pt | Estimate | SE | P-values  Satterthwaite |
| 5em8 | 0.891 | 6.585 | 0.892 |
| 5em7 | 2.550 | 6.179 | 0.680 |
| 5em6 | 2.009 | 5.370 | 0.708 |
| 5em5 | -0.768 | 4.444 | 0.863 |
| 5em4 | 0.930 | 3.225 | 0.773 |
| 5em3 | -0.577 | 1.697 | 0.734 |
| 0p05 | -0.086 | 0.842 | 0.918 |
| 0p1 | 0.078 | 0.694 | 0.911 |
| 0p2 | 0.141 | 0.587 | 0.810 |
| 0p3 | 0.288 | 0.551 | 0.601 |
| 0p4 | 0.177 | 0.534 | 0.740 |
| 0p5 | 0.231 | 0.526 | 0.661 |
| **Change in MHI-3** | | | |
| pt | Estimate | SE | P-values  Satterthwaite |
| 5em8 | 0.612 | 0.294 | 0.038 |
| 5em7 | 0.588 | 0.275 | 0.033 |
| 5em6 | 0.471 | 0.239 | 0.050 |
| 5em5 | 0.261 | 0.199 | 0.189 |
| 5em4 | 0.093 | 0.145 | 0.518 |
| 5em3 | -0.055 | 0.076 | 0.470 |
| 0p05 | -0.047 | 0.038 | 0.212 |
| 0p1 | -0.023 | 0.031 | 0.467 |
| 0p2 | -0.012 | 0.026 | 0.647 |
| 0p3 | -0.008 | 0.025 | 0.762 |
| 0p4 | -0.007 | 0.024 | 0.756 |
| 0p5 | -0.008 | 0.024 | 0.748 |

Table S43. Results linear mixed models PRS analysis: association between polygenic risk scores for cigarettes per day and change in symptoms of depression using the SF-36 subscale score and MHI-3 score (corrected for age and gender) in smokers.

*Fixed effects in the models were polygenic risk score for cigarettes per day, PC1, PC2, PC3, age, gender and time. Random effects were by-subject random slopes for time and intercepts for respondents. Data are estimates and P-values were calculated using the Satterthwaite approach and adjusted for multiple testing using the Benjamini & Hochberg method.*

PRS CPD on change in SF-36 and change in MHI-3 *(non-smokers)*

| **Change in SF-36** | | | |
| --- | --- | --- | --- |
| pt | Estimate | SE | P-values  Satterthwaite |
| 5em8 | 2.659 | 2.981 | 0.373 |
| 5em7 | 2.382 | 2.818 | 0.398 |
| 5em6 | 2.239 | 2.520 | 0.375 |
| 5em5 | 0.249 | 2.047 | 0.903 |
| 5em4 | -0.075 | 1.440 | 0.959 |
| 5em3 | 0.042 | 0.793 | 0.958 |
| 0p05 | -0.081 | 0.390 | 0.836 |
| 0p1 | -0.347 | 0.323 | 0.283 |
| 0p2 | -0.337 | 0.275 | 0.220 |
| 0p3 | -0.232 | 0.258 | 0.369 |
| 0p4 | -0.140 | 0.249 | 0.575 |
| 0p5 | -0.171 | 0.245 | 0.486 |
| **Change in MHI-3** | | | |
| pt | Estimate | SE | P-values  Satterthwaite |
| 5em8 | -0.035 | 0.130 | 0.790 |
| 5em7 | -0.019 | 0.123 | 0.879 |
| 5em6 | -0.025 | 0.110 | 0.818 |
| 5em5 | -0.064 | 0.089 | 0.474 |
| 5em4 | -0.107 | 0.063 | 0.088 |
| 5em3 | -0.078 | 0.034 | 0.024 |
| 0p05 | -0.011 | 0.017 | 0.506 |
| 0p1 | -0.013 | 0.014 | 0.359 |
| 0p2 | -0.011 | 0.012 | 0.347 |
| 0p3 | -0.011 | 0.011 | 0.342 |
| 0p4 | -0.008 | 0.011 | 0.441 |
| 0p5 | -0.008 | 0.011 | 0.442 |

Table S44. Results linear mixed models PRS analysis: association between polygenic risk scores for cigarettes per day and change in symptoms of depression using the SF-36 subscale score and MHI-3 score (corrected for age and gender) in non-smokers.

*Fixed effects in the models were polygenic risk score for cigarettes per day, PC1, PC2, PC3, age, gender and time. Random effects were by-subject random slopes for time and intercepts for respondents. Data are estimates and P-values were calculated using the Satterthwaite approach and adjusted for multiple testing using the Benjamini & Hochberg method.*

PRS DPW on change in SF-36 and change in MHI-3

| **Change in SF-36** | | | |
| --- | --- | --- | --- |
| pt | Estimate | SE | P-values  Satterthwaite |
| 5em8 | 7.728 | 4.512 | 0.087 |
| 5em7 | 5.531 | 3.753 | 0.141 |
| 5em6 | 3.668 | 3.119 | 0.240 |
| 5em5 | 2.482 | 2.408 | 0.307 |
| 5em4 | 2.222 | 1.637 | 0.175 |
| 5em3 | 0.624 | 0.935 | 0.505 |
| 0p05 | -0.526 | 0.475 | 0.268 |
| 0p1 | -0.332 | 0.397 | 0.403 |
| 0p2 | -0.436 | 0.343 | 0.204 |
| 0p3 | -0.355 | 0.323 | 0.272 |
| 0p4 | -0.416 | 0.313 | 0.185 |
| 0p5 | -0.439 | 0.309 | 0.156 |
| **Change in MHI-3** | | | |
| pt | Estimate | SE | P-values  Satterthwaite |
| 5em8 | 0.301 | 0.200 | 0.133 |
| 5em7 | 0.254 | 0.166 | 0.127 |
| 5em6 | 0.190 | 0.138 | 1.700 |
| 5em5 | 0.125 | 0.107 | 0.240 |
| 5em4 | 0.052 | 0.073 | 0.474 |
| 5em3 | -0.002 | 0.041 | 0.967 |
| 0p05 | -0.027 | 0.021 | 0.192 |
| 0p1 | -0.010 | 0.018 | 0.560 |
| 0p2 | -0.009 | 0.015 | 0.547 |
| 0p3 | -0.005 | 0.014 | 0.749 |
| 0p4 | -0.006 | 0.014 | 0.687 |
| 0p5 | -0.007 | 0.014 | 0.607 |

Table S45. Results linear mixed models PRS analysis: association between polygenic risk scores for drinks per week and change in symptoms of depression using the SF-36 subscale score and MHI-3 score (corrected for age and gender).

*Fixed effects in the models were polygenic risk score for drinks per week, PC1, PC2, PC3, age, gender and time. Random effects were by-*subject *random slopes for time and intercepts for respondents. Data are estimates and P-values were calculated using the Satterthwaite approach and adjusted for multiple testing using the Benjamini & Hochberg method.*

PRS SI on change in SF-36 and change in MHI-3

| **Change in SF-36** | | | |
| --- | --- | --- | --- |
| pt | Estimate | SE | P-values  Satterthwaite |
| 5em8 | 3.769 | 5.437 | 0.488 |
| 5em7 | 3.318 | 4.317 | 0.442 |
| 5em6 | 3.455 | 3.092 | 0.264 |
| 5em5 | 0.059 | 2.287 | 0.979 |
| 5em4 | 1.201 | 1.503 | 0.424 |
| 5em3 | 0.280 | 0.832 | 0.737 |
| 0p05 | -0.273 | 0.455 | 0.549 |
| 0p1 | -0.051 | 0.391 | 0.897 |
| 0p2 | -0.145 | 0.343 | 0.674 |
| 0p3 | -0.044 | 0.325 | 0.893 |
| 0p4 | -0.007 | 0.316 | 0.982 |
| 0p5 | -0.004 | 0.311 | 0.990 |
| **Change in MHI-3** | | | |
| pt | Estimate | SE | P-values  Satterthwaite |
| 5em8 | 0.153 | 0.241 | 0.526 |
| 5em7 | 0.059 | 0.191 | 0.759 |
| 5em6 | 0.165 | 0.137 | 0.228 |
| 5em5 | 0.117 | 0.101 | 0.248 |
| 5em4 | 0.129 | 0.066 | 0.053 |
| 5em3 | 0.021 | 0.037 | 0.571 |
| 0p05 | -0.018 | 0.020 | 0.372 |
| 0p1 | -0.014 | 0.017 | 0.428 |
| 0p2 | -0.009 | 0.015 | 0.576 |
| 0p3 | -0.005 | 0.014 | 0.706 |
| 0p4 | -0.006 | 0.014 | 0.676 |
| 0p5 | -0.004 | 0.014 | 0.795 |

Table S46. Results linear mixed models PRS analysis: association between polygenic risk scores for smoking initiation and change in symptoms of depression using the SF-36 subscale score and MHI-3 score (corrected for age and gender).

*Fixed effects in the models were polygenic risk score for smoking initiation, PC1, PC2, PC3, age, gender and time. Random effects were by-subject random slopes for time and intercepts for respondents. Data are estimates and P-values were calculated using the Satterthwaite approach and adjusted for multiple testing using the Benjamini & Hochberg method.*

**Removal of LD regions from PRS analysis**

| Chromosome | Base pair position  (start point to end point) |
| --- | --- |
| 1 | 48000000-52000000 |
| 2 | 86000000-100500000 |
| 2 | 183000000-190000000 |
| 3 | 47500000-50000000 |
| 3 | 83500000-87000000 |
| 5 | 44500000-50500000 |
| 5 | 129000000-132000000 |
| 6 | 25500000-33500000 |
| 6 | 57000000-64000000 |
| 6 | 140000000-142500000 |
| 7 | 55000000-66000000 |
| 8 | 8000000-12000000 |
| 8 | 43000000-50000000 |
| 8 | 112000000-115000000 |
| 10 | 37000000-43000000 |
| 11 | 87500000-90500000 |
| 12 | 33000000-40000000 |
| 20 | 32000000-34500000 |
| 8 | 8135000-12000000 |
| 17 | 40900000-45000000 |

Table S47. 20 complex-LD regions and long-range LD regions that were excluded from PRS analysis(Price et al., 2008)

**References**

Bos, E. H., ten Have, M., van Dorsselaer, S., Jeronimus, B. F., de Graaf, R., & de Jonge, P. (2018). Functioning before and after a major depressive episode: pre-existing vulnerability or scar? A prospective three-wave population-based study. *Psychological Medicine*, *48*(13), 2264–2272. https://doi.org/10.1017/S0033291717003798

Choi, S. W., Mak, T. S.-H., & O’Reilly, P. F. (2020). Tutorial: a guide to performing polygenic risk score analyses. *Nature Protocols*, *15*(9), 2759–2772. https://doi.org/10.1038/s41596-020-0353-1

Euesden, J., Lewis, C. M., & O’Reilly, P. F. (2015). PRSice: Polygenic Risk Score software. *Bioinformatics*, *31*(9), 1466–1468. https://doi.org/10.1093/bioinformatics/btu848

European Network of National Networks studying Gene-Environment Interactions in Schizophrenia (EU-GEI), van Os, J., Rutten, B. P., Myin-Germeys, I., Delespaul, P., Viechtbauer, W., … Mirjanic, T. (2014). Identifying gene-environment interactions in schizophrenia: contemporary challenges for integrated, large-scale investigations. *Schizophrenia Bulletin*, *40*(4), 729–736. https://doi.org/10.1093/schbul/sbu069

*HapMap 3 - Wellcome Sanger Institute*. (2021). Retrieved from https://www.sanger.ac.uk/resources/downloads/human/hapmap3.html Accessed 28 May 2019.

Liu, M., Jiang, Y., Wedow, R., Li, Y., Brazel, D. M., Chen, F., … Vrieze, S. (2019). Association studies of up to 1.2 million individuals yield new insights into the genetic etiology of tobacco and alcohol use. *Nature Genetics*, *51*(2), 237–244. https://doi.org/10.1038/s41588-018-0307-5

McHorney, C. A., Ware, J. E., Rachel Lu, J. F., & Sherbourne, C. D. (1994). The MOS 36-ltem Short-Form Health Survey (SF-36): III. Tests of Data Quality, Scaling Assumptions, and Reliability Across Diverse Patient Groups. *Medical Care*, *32*(1), 40–66. https://doi.org/10.1097/00005650-199401000-00004

Price, A. L., Patterson, N. J., Plenge, R. M., Weinblatt, M. E., Shadick, N. A., & Reich, D. (2006). Principal components analysis corrects for stratification in genome-wide association studies. *Nature Genetics*, *38*(8), 904–909. https://doi.org/10.1038/ng1847

Price, A. L., Weale, M. E., Patterson, N., Myers, S. R., Need, A. C., Shianna, K. V, … Reich, D. (2008). Long-range LD can confound genome scans in admixed populations. *American Journal of Human Genetics*, *83*(1), 132–135; author reply 135-9. https://doi.org/10.1016/j.ajhg.2008.06.005

Purcell, S., Neale, B., Todd-Brown, K., Thomas, L., Ferreira, M. A. R., Bender, D., … Sham, P. C. (2007). PLINK: a tool set for whole-genome association and population-based linkage analyses. *American Journal of Human Genetics*, *81*(3), 559–575. https://doi.org/10.1086/519795

Walters, R. K., Polimanti, R., Johnson, E. C., McClintick, J. N., Adams, M. J., Adkins, A. E., … Agrawal, A. (2018). Transancestral GWAS of alcohol dependence reveals common genetic underpinnings with psychiatric disorders. *Nature Neuroscience*, *21*(12), 1656–1669. https://doi.org/10.1038/s41593-018-0275-1
